# The impact of eHealth on relationships and trust in primary care: a review of reviews

**DOI:** 10.1186/s12875-023-02176-5

**Published:** 2023-11-03

**Authors:** Meena Ramachandran, Christopher Brinton, David Wiljer, Ross Upshur, Carolyn Steele Gray

**Affiliations:** 1 Bridgepoint Collaboratory for Research and Innovation, Lunenfeld-Tanenbaum Research Institute, Sinai Health, 1 Bridgepoint Dr, Toronto, ON M4M 2B5 Canada; 2 School of Physical and Occupational Therapy, McGill University, 3654 Promenade Sir-William-Osler, Montreal, QC H3G 1Y5 Canada; 3 Michael G. DeGroote School of Medicine, McMaster University, 1280 Main Street West, Hamilton, ON L8S 4L8 Canada; 4 Education Technology Innovation, University Health Network, 190 Elizabeth St, Toronto, ON M5G 2C4 Canada; 5 Department of Psychiatry, University of Toronto, 155 College St, Toronto, ON M5T 3M6 Canada; 6 Institute for Health Policy, Management and Evaluation, University of Toronto, 155 College St, Toronto, ON M5T 3M6 Canada; 7 Centre for Addiction and Mental Health, 1000 Queen St W, Toronto, ON M6J 1H4 Canada; 8 Dalla Lana School of Public Health, University of Toronto, 155 College St, Toronto, ON M5T 3M6 Canada

**Keywords:** eHealth, Technology, Primary care, Relationships, Trust

## Abstract

**Background:**

Given the increasing integration of digital health technologies in team-based primary care, this review aimed at understanding the impact of eHealth on patient-provider and provider-provider relationships.

**Methods:**

A review of reviews was conducted on three databases to identify papers published in English from 2008 onwards. The impact of different types of eHealth on relationships and trust and the factors influencing the impact were thematically analyzed.

**Results:**

A total of 79 reviews were included. Patient-provider relationships were discussed more frequently as compared to provider-provider relationships. Communication systems like telemedicine were the most discussed type of technology. eHealth was found to have both positive and negative impacts on relationships and/or trust. This impact was influenced by a range of patient-related, provider-related, technology-related, and organizational factors, such as patient sociodemographics, provider communication skills, technology design, and organizational technology implementation, respectively.

**Conclusions:**

Recommendations are provided for effective and equitable technology selection, application, and training to optimize the impact of eHealth on relationships and trust. The review findings can inform providers’ and policymakers’ decision-making around the use of eHealth in primary care delivery to facilitate relationship-building.

**Supplementary Information:**

The online version contains supplementary material available at 10.1186/s12875-023-02176-5.

## Background

Primary care is a person’s first point of contact in healthcare systems and includes “disease prevention, health promotion, population health, and community development” ([1, 2] p1). Primary care across the globe is shifting towards team-based models that bring together interprofessional teams of family physicians, nurse practitioners, registered nurses, social workers, dietitians, and other professionals to provide holistic and comprehensive care [3–6]. These models are designed to address the needs of individuals with multimorbidity and complex conditions in the community as they can offer a diverse skill set to meet the variable needs of this population [7]. Along with an evolution towards team-based primary care models, this past decade has also witnessed an increasing global interest and rapid uptake of digital health in primary care [8–10], hastened by the COVID-19 pandemic [11, 12]. Some jurisdictions are considering a “digital-first” primary care model where technology is used as the default care delivery mechanism [13], while others have noted a need to balance appropriate and equitable hybrid care delivery [10].

Digital health broadly refers to the use of technologies for health [14]. Technologies include information and communication technology (also referred to as eHealth), which includes the use of mobile wireless technologies (often referred to as mHealth as a specific type of eHealth) [14]. Digital health technologies can also include emerging technologies, processes, and platforms like big data, genomics, machine learning, and artificial intelligence [14]. eHealth includes: (i) management systems; (ii) communication systems; (iii) computerised decision support systems; and (iv) information systems [15]. The implementation and effectiveness of eHealth is influenced by a complex array of factors and can impact several facets of care delivery [16].

One aspect that can potentially be altered is the nature of relationships and trust between patients and their providers, and within provider teams. Relationships between patients and providers, built on trust, knowledge, regard, and loyalty, have been demonstrated to be fundamental to healthcare delivery [17]. This is particularly important in primary care where patients will tend to have longer-term relationships with their provider or practice [18]. Strong trust-based relationships between providers within teams can enable a positive work environment, improved communication, effective teamwork, and care coordination [19, 20].

### eHealth and patient-provider relationships

Patient-provider relationships are often referred to using terms like therapeutic relationship, therapeutic alliance, communication, interaction, and rapport [21–27]. Trust is thought to be an important component of this relationship [28] and its development has been found to require multiple interactions over time [29]. Promoting trust in the patient-provider relationship includes the demonstration of three key provider attributes: interpersonal and technical competence, moral comportment, and vigilance [30]. Patients perceive trust in providers as linked to their active participation and satisfaction with care [31, 32]. An absence of trust in providers is associated with reductions in treatment adherence and care seeking behaviours by patients, and reduced continuity of care [33] (i.e., connected and coordinated care while moving through the healthcare system) [34].

Trust-based patient-provider relationships are changing with the expansion of eHealth. Henson et al. use the term ‘digital therapeutic alliance’ to refer to patient-provider relationships established through mental health apps [35]. The interconnection between technology and therapeutic relationships is evident in Mesko and Győrffy’s ([36] p2) definition of digital health as “the cultural transformation of how disruptive technologies that provide digital and objective data accessible to both health care providers and patients leads to an equal-level doctor-patient relationship with shared decision-making and the democratization of care”. Studies have reported positive changes accompanying this transformation. Patients may experience greater empowerment through improved access to health information and resources and can assume a more active role in communication and decision-making [36–38]. Providers may experience shifts towards empathy-driven care [39], assume the role of a guide to direct patients towards high-quality information and services [36], and support active patient engagement with technology [40]. Some providers value the use of technology for prioritizing patient values, enabling patient autonomy [41], and making caregivers part of the team [42].

However, the impact of technology on relationships has also been termed “a double-edged sword” with significant ethical and safety implications [38]. Technology is thought to harm the relationship and reduce efficiency if patients obtain irrelevant information or misinterpret information [37, 38]. ( For instance, patients may misinterpret data or test results accessed through technology such as self-monitoring devices and smartphone apps when the provider’s involvement is limited) [37]. Patients may also access information through resources on the Internet that may enable them in engage actively in dialogue with the provider but may also lead to them obtaining irrelevant or inaccurate information. Some providers have expressed concerns related to overuse of technology by patients and caregivers (e.g., frequently checking blood sugar or pressure when deemed unnecessary by the provider) [42] and technology taking their attention away from patients during the clinical encounter [41].

### eHealth and provider-provider relationships

Relationships between primary care providers that “provide support and sustenance” are among the key factors for compassion among healthcare workers ([43] p123). Like the case of patient-provider relationships, trust is integral to strong team relationships and can contribute to better quality of care and practice improvement through open discussions of successes and failures among team members [23]. In an increasingly virtual care delivery environment, trust-based relationships between providers can facilitate interprofessional collaboration [44]. Interpersonal trust has been identified as a primary determinant of performance in virtual relationships between telemedicine providers [45]. A lack of trust between telehealth nurses and other primary care professionals was found to create tensions in their relationships [37]. The use of health information technology can enhance trust between providers when it facilitates reviewing and affirming non-physician clinicians’ decisions or erode trust when it limits opportunities for developing familiarity and comfort [25].

### Objectives and approach

While there is a growing body of literature on the impact of eHealth on patient-provider and provider-provider relationships and trust in primary care, questions remain around how to best integrate eHealth into primary health care systems to facilitate relationship-centred care and uphold the “humanness” of primary care [46]. There is a need to examine this issue to generate specific information that can inform decision- and policymaking around the integration and implementation of eHealth into primary care while considering its impact on relationships and trust.

This paper reports on a review of reviews [47] to synthesise high-level evidence on relationships and trust as related to the use of eHealth in primary care. This approach was selected to identify what is currently known and unknown in this field by summarizing evidence from the large number of existing evidence syntheses, and to generate recommendations on how to ensure eHealth adoption permits and strengthens relationships and trust in primary care. To guide the review, we sought to answer the research question: *How does eHealth impact patient-provider and provider-provider relationships and trust in primary care?*Given the importance of health equity, especially in relation to the use of digital health in primary care [48], we also sought to understand if eHealth has a differential impact on trust and relationships across different groups (e.g., sociodemographic groups).

## Methods

### Search strategy

The search strategy was developed for Medline and adapted to EMBASE and Cochrane databases (Additional file 1). Four concepts were included: ‘*primary care’, ‘digital health technologies’, ‘relationships’,* and ‘*trust’.* Strategies developed for previous reviews with a librarian’s assistance helped build the search for ‘*primary care’* and *‘digital health technologies’*. A strategy was developed for the other two concepts (i.e., *‘relationships’* and *‘trust’*) using subject headings and non-indexed keywords identified through team brainstorming and literature scans. The initial search was conducted in May 2021, followed by an updated search using the same strategy in June 2022.

### Inclusion criteria and study selection

The search focused on peer-reviewed evidence syntheses published in English from 2008 onwards. This timeline was determined based on trends noted in two reviews on digital health in primary care that indicated that most papers were published after 2008 [49, 50]. Included reviews (i) were located in a primary care setting, either exclusively or along with other settings (ii) discussed patient-provider and/or provider-provider relationships and/or trust, and (iii) included the use of digital health/eHealth/mHealth technologies (as defined above, and as consistent with our search criteria listed in search lines 10–25 in Additional file 1) allowing for interaction or information-sharing between patients and providers and/or between providers. As the focus of the review was on adult patients receiving primary care services, reviews exclusively discussing patients below 18 years of age were excluded. Primary empirical studies, conference abstracts, editorials and grey literature were also excluded.

**Fig. 1:**
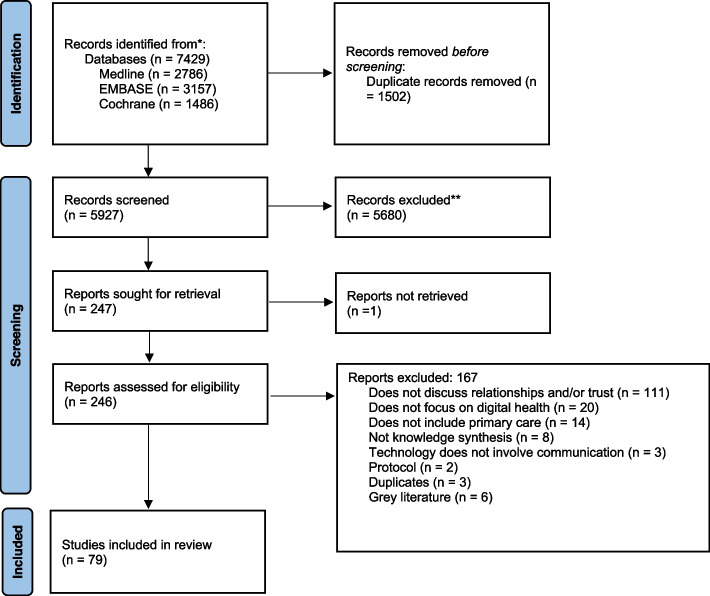
PRISMA chart

The search results were validated using five articles chosen by the research team that met the inclusion criteria. Articles were then uploaded to EndNote reference manager to remove duplicates, and then transferred to Covidence review management platform for screening. The Preferred Reporting Items for Systematic Reviews and Meta-Analyses (PRISMA) flow diagram (Fig. 1) depicts the study selection process. Text screening followed two phases: 1) title and abstract and 2) full text.

1. Title and abstract screening: Two rounds of title and abstract screening tests between three team members were conducted to ensure agreement and alignment with the inclusion criteria at this stage. All three members screened a random sample of 100 titles and abstracts to check if they met the inclusion criteria. Cohen’s Kappa values [51, 52] were calculated between pairs of reviewers (e.g. Rev 1-Rev2; Rev 2-Rev3; Rev 1- Rev3) resulting in Kappa values ranging from 0.496 to 0.754, suggesting moderate to substantial agreement by the second round. Team meetings were held to discuss conflicts, and after the second round it was determined that all three reviewers had come to a common understanding of the inclusion/exclusion criteria to proceed with a single-reviewer approach.
2. Full-text screening: At the stage of full-text screening a single-reviewer approach was deemed sufficient due to clear understanding of inclusion and exclusion criteria established by the reviewers, and due to time and resource constraints..

### Data extraction and synthesis

Three members of the research team conducted data extraction. A data extraction sheet was developed for this study and piloted on three articles. It included: type of review; number of studies; research paradigm of authors (e.g., postpositivist, constructionist); study aims; participants; settings; type(s) of technology; definitions of relationships and trust and/or connected terms; factors influencing impact of eHealth on relationships and/or trust; and any discussions around equity (how this impact might differ in different groups).

Based on definitions of relationships from our preliminary literature searches [21–27], we included reviews directly referring to ‘relationships’ or using other related terms like ‘collaboration’, ‘communication’, ‘connectedness/connection’, ‘interaction’, ‘empathy’, ‘respect’, and ‘understanding’. We searched each included review to see how they had described these terms and then aggregated and analysed these descriptions to identify patterns and interrelationships between terms. We also searched each review for descriptions of the impact of eHealth on relationships and/or trust and classified the impact as positive, negative, or mixed (both positive and negative). When the type of impact was not directly mentioned by the authors, two members of the research team classified the impact based on their interpretations of the authors’ descriptions and following discussions with each other. Technologies were classified using Mair et al.’s four eHealth domains described in Table 1 [15, 53]. Thematic analysis was conducted to determine the impact of different types of eHealth on relationships and/or trust and any influencing factors. Two members of the research team coded data from each article on influencing factors separately. Coding involved highlighting and labelling relevant sections from the extracted data in a Word document. Both members then met to discuss and merge the developed codes into a single document. One member then analysed these codes, and four broad categories were developed (patient-related, provider-related, technology-related, and other factors). The second member then reviewed these categories by checking if they aligned with data extracted from 10 reviews.

**Table 1:** Classification of eHealth

Category	Definition
Management systems	“…allow for the acquisition, storage, transmission, and display of administrative or clinical activities related to patients, such as Electronic Health Records (EHRs) or Electronic Medical Records (EMRs).”
Communication systems	“…can be used for diagnostic, management, counseling, educational or support purposes” and “can be implemented to facilitate communication between health professionals or between health professionals and patients”, like email, mobile phones, telemedicine.
Computerized Decision Support Systems (CDSSs)	“…automated systems accessible from various devices, such as computer, mobile phone, or personal digital assistants (PDAs)” that “support decision-making for health professionals and assist them in practicing within clinical guidelines and care pathways”.
Information systems	“…refer to the use of Internet technology to access health-related information sources” like web-based resources and eHealth portals.

Source: Mair et al. [15] as cited in Rouleau et al. [53]

## Results

### Overview of reviews

**Fig. 2:**
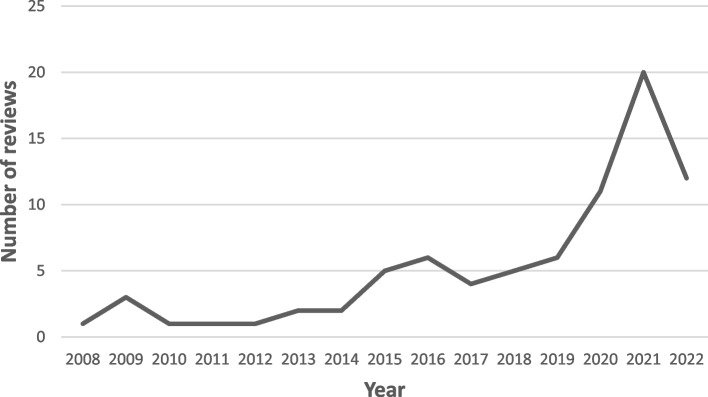
Number of reviews by year Note: As the updated search was conducted in June 2022, the number of reviews in 2022 only includes those conducted between January and June

The screening process yielded a total of 79 reviews were included (55 from the initial search and 24 from the updated search). Most reviews were published from 2015 onwards with a notable increase in numbers in 2020, 2021, and 2022 (Fig. 2). Most reviews focused on patient-provider relationships and/or trust (76 of 79), three reviews only discussed provider-provider relationships and/or trust, and 19 reviews focused on both groups. The majority of reviews either focused exclusively on adult patient populations (31 of 79) and providers from multiple disciplines (37 of 79) or did not describe the patient (37 of 79) and provider population (35 of 79). Reviews either exclusively focused on primary care (14 of 79), discussed a range of settings including primary care (40 of 79), or did not clearly describe the settings (25 of 79). Of the four domains of eHealth technology, communication systems were discussed most frequently (38 of 79), followed by reviews discussing multiple types of technology across the four domains (19 of 79) and management systems (17 of 39). Fourteen reviews discussed how the impact of eHealth (mostly communication systems) on patient-provider relationships and/or trust may differ based on age, socioeconomic status, functional ability, language, or being part of a minority/disadvantaged group [16, 54–66].

Seventeen reviews discussed the impact of COVID-19 pandemic [56–58, 63, 64, 67–78]. Eight reviews described the role of the pandemic in facilitating a rapid shift towards the increased use of digital health in the background or discussion sections, mostly to justify the need for their review [56, 67, 68, 70–72, 74, 75]. Seven reviews mentioned including studies related to the COVID-19 pandemic and factored this into their analysis [57, 64, 69, 73, 76–78] to understand things like feasibility of implementation of digital health [64] but did not conduct any analyses related to the impact of digital health on relationships and/or trust. Only two reviews specifically focused on the use of telemental health [58] and remote consultations [63] during the pandemic and reported some positive and negative impacts of these types of technology on patient-provider relationships.

Eight reviews directly examined relationships and/or trust in the context of eHealth [59, 79–85]. Eleven reviews examined related concepts like communication, interaction, and therapeutic alliance in an eHealth context [61, 62, 86–94]. In all other reviews, relationships and/or trust were not the focus but were discussed along with other findings. Tables 2 and 3 outline the characteristics of the included reviews.

**Table 2:** Review characteristics

**Characteristics**	**Number of reviews**
***Type of relationship discussed***	
Only patient-provider relationships	57
Only provider-provider relationships	3
Patient-provider and provider-provider relationships	19
***Setting***	
Primary care only	14
Multiple settings including primary care	40
Not described^a^	25
***Patient population***	
Adult only	31
Mixed	11
Not described	37
***Provider population***	
Multiple disciplines	37
Single discipline	7
Not described	35
***Types of technology***	
Management systems	17
Communication systems	38
Computerized Decision Support systems	3
Information systems	2
Papers that discussed multiple technology types	19
***Discussions on equity***	
Discussed directly relating to patient-provider relationships and/or trust	14
Discussed directly relating to provider-provider relationships and/or trust	0
Not discussed	65
***Discussions on the impact of COVID-19***	
Discussed directly relating to patient-provider relationships and/or trust	2
Discussed directly relating to provider-provider relationships and/or trust	0
Discussed in another context	15

^a^This category refers to reviews that did not explicitly mention a focus on primary care in their inclusion criteria. However, we interpreted these reviews as focusing largely on primary care based on the way their introduction/background sections were framed or based on their descriptions of the included studies. E.g., Tapuria et al. [95] do not mention ‘primary care’ in their inclusion criteria but their discussion of doctor-patient relationships and trust draws largely on examples of primary care physicians and their patients

**Table 3:** Description of reviews

**Review**	**Type of review**	**Type of technology**	**Setting**	**Description of patients**	**Description of providers**
***Management systems***					
Shachak et al. (2009) [92]	Literature review	Electronic Medical Records and Computer Physician Order Entry systems	Primary care clinics	Not described	Physicians (general practitioners, family medicine, internal medicine residents, ER doctors), faculty internists, nurses
Irani et al. (2009) [96]	Systematic review	Electronic Health Records used in the examination room	Ambulatory care, outpatient office settings	Not described for all studies, one study included patients with hypertension and dyslipidemia, another included paediatric patients	Physicians
McGinn et al. (2011) [97]	Systematic review	Electronic Health Records, Electronic Medical Records, computerized patient information systems and medical records, personal health records, portable computers, smart card and summary care records	Not described	Not described	Mostly physicians, nurses, less commonly pharmacists, midwives, social workers
Bassi et al. (2012) [98]	Systematic review	Information Communication Technologies (including Electronic Medical Records and other types like computer-based information systems, computerized claims or billing systems, computerized scheduling or prescribing systems)	Ambulatory physician office practices (including primary care settings)	Not described	Family physicians/general practitioners, specialists including ophthalmologists pediatricians, internal medicine, obstetrics and gynecology, physician assistants, nurses, nurse practitioners
Kazmi et al. (2013) [89]	Systematic review	Electronic Health Records	Outpatient settings	Not described	Physicians
Nguyen et al. (2014) [99]	Systematic literature review	Electronic Health Records	Primary care, secondary care, tertiary care, ambulatory care, long-term care, community and consumer based, cross-sectional (spanning over one care level)	Not described	Doctors and nurses
Alkureishi et al. (2015) [79]	Systematic review	Electronic Medical Records	Mostly outpatient primary care settings, some in specialty clinic and inpatient settings	Mostly adult and some paediatric patients	Not described
Rathert et al. (2017) [91]	Systematic review	Electronic Health Records and Electronic Medical Records (referred to together as EHR)	Any health service setting	Adult patients excluding dental or psychiatric patients, participants not described	Physicians
Mold et al. (2018) [100]	Systematic review	Computerized Medical Records and online services	Primary care	Adult patients with Type 2 Diabetes Mellitus	Not described
Wisner et al. (2019) [101]	Integrative review	Electronic Health Records	Hospital settings including mostly inpatient acute care units	Not described	Mostly registered nurses, also included physicians and midwives
Diffin et al. (2019) [102]	Systematic realist review	Patient Personal Health Records	Children’s hospital, disease specific outpatient clinics and departments within hospitals, primary care, rehabilitation hospital, special education schools/units, not-for-profit organizations	Children and young persons (0–24 years) including adolescents with intellectual disability, depressive symptoms, complex health and palliative needs, cystic fibrosis, diabetes mellitus, juvenile idiopathic arthritis, asthma, autism spectrum diagnosis	Not described
Lordon et al. (2020) [81]	Systematic review	Patient Generated Health Data	Mostly primary care, one paper focused on surgical setting	Not described	Not described
Benjamins et al. (2021) [66]	Scoping review	Patient-Accessible Electronic Health Records	Varied settings, including hospitals and primary care	Broad range of adult patients, with some papers focusing on specific patient groups including cancer, cardiac, chronically ill, HIV-positive, psychiatric, gynecologic patients and veterans	Not described
Tapuria et al. (2021) [95]	Systematic review	Electronic Health Records that could be accessed by patients through patient portals	Not described	Not described	Not described
Wark et al. (2021) [60]	Scoping review	Electronic Health Records	Not described	Not described	Not described
Zurynski et al. (2021) [103]	Scoping review	Electronic Medical Records	Primary studies were conducted in different settings including psychiatric hospitals, mental health facilities, psychiatry services, primary care clinics, child and youth psychiatry clinics, community mental health clinics One review focused on mental health settings, others focused on other health settings	Not described	Mental health professionals including psychiatrists, psychologists, nurses, and other professionals invovled in treating people with mental health disorders including allied health professionals
Schwarz et al. (2022) [104]	Scoping review	Patient-accessible electronic health records (PAEHR)	Inpatient and outpatient mental health settings and primary care settings	Service users with mental health conditions, veterans	Healthcare professionals (including mental health professionals like psychologists, social workers and nurses, general medical practitioners)
***Communication systems***					
Verhoeven et al. (2010) [105]	Systematic review	Asychronous (most studies) or synchronous teleconsultation or a combination of both	Primary, secondary, tertiary and integrated care settings	Patients with diabetes	Specialized nurses, other specialists, primary care providers, case managers or professional role not specified
Brewster et al. (2013) [106]	Systematic review	Technology to remotely fulfill healthcare needs (including video-phone links and remote monitoring equipment)	Not described	Patients with COPD and CHF	Frontline staff including general practitioners, cardiologists, nurses, hospital staff, social care staff,
Simpson et al. (2014) [62]	Systematic review	Videoconferencing	Not described	Adult, adolescent, and child clients with wide range of mental health disorders	Psychologists
Nguyen et al. (2015) [90]	Systematic review	Paging systems, web-based smartphone messaging systems, smartphone-linked email system, wireless email, blog, hands-free communication device	Hospitals and clinics	Not described	Trainees, nurses, attending physicians, pharmacists, medical teams, anesthesiologists
Keijser et al. (2016) [107]	Systematic review	Health Information Technology (including phone, text messaging, email and teleconferencing)	Integrated healthcare (e.g., chronic disease management; home telecare programs; multidisciplinary team consultations; virtual integrated practice), thematic collaborations (e.g. healthcare quality improvement initiatives; communities of practice) and patient-centred online programs	Not described	Not described
Petit et al. (2016) [108]	Scoping review	Smart devices	Primary care (preventative care)	Not described	Not described
Henry et al. (2017) [65]	Systematic literature review	Telecommunications technologies (including phone, videophone/videoconferencing, telemonitoring, computers, electronic communication and robots)	Home care, primary and specialist care, mental health/counseling, multi-site teams, and educational settings	Not described	Healthcare providers, students, and trainees
Richards et al. (2018) [109]	Systematic review	Mobile phones, tablets, Personal Digital Assistants, handheld devices	Non-inpatient settings or non-patient and inpatient settings	Patients with cancer (mostly adults, but also included children and adolescents)	Not described
Watkins et al. (2018) [110]	Realist review	mHealth	All types of low-resourced settings in LMICs	Patients over 18 years of age with chronic diseases, including stroke, hypertension and HIV	Community health workers, nurses, doctors
Penny et al. (2018) [111]	Systematic review	Telehealth through videoconferencing (one study also involved remote monitoring and another involved a web forum)	Varied settings including NICUs, chemotherapy, hospitals, hospice, home care, nursing homes, clinics, medical centres, outpatient paediatric clinics	Varied populations including paediatric (NICU, suspected child abuse) and adult populations (hospice, COPD, postpartum parents)	Registered nurses and midwives
Walker et al. (2019) [112]	Systematic review	Remote monitoring	Not described, but study only focused on telemonitoring for non-hospitalized patients	Patients over 18 years of age with chronic health conditions, including COPD, heart failure, diabetes, end stage kidney disease and hypertension	Not described
Qudah et al. (2019) [83]	Systematic review	mHealth apps available on smartphones or personal digital assistants	Not described	Adolescent and adult patients with mental health conditions, diabetes, cancer, pregnancy, respiratory disorders, postsurgical care and other conditions	Physicians, nurses, multidisciplinary teams, and other providers
Foong et al. (2020) [113]	Systematic review	Telemedicine, mHealth and specific mHealth app	Clinical or community settings	Patients with diabetic foot ulcer	Nurses, physicians, podiatrists, physician assistants
Odendaal et al. (2020) [16]	Qualitative evidence synthesis	mHealth	Primary care settings including clients’ homes, clinics, hospitals, general practices and schools	Not described	All healthcare workers, including lay, paraprofessional, and professional workers (nurses, paramedics, doctors, midwives, pharmacists, and lab staff)
Vimalananda et al. (2020) [114]	Systematic review	e-consults	Primary care physician consultations, medical centres, tertiary academic medical centre, integrated health delivery systems, community health centres, veteran's affairs, state/city/regional/district programs, non-governmental organizations, private company	Not described	Primary care providers and specialists
Wehmann et al. (2020) [93]	Systematic review	Email, web-based programs, telephone, and a smartphone app	Not described	Patients aged over 18 years diagnosed with unipolar depression	Therapists including Masters students and licensed psychotherapists and psychologists
Wickramasekera et al. (2020) [115]	Systematic review	Patient-completed electronic clinical assessment tool	Secondary care settings including tertiary care centre, medical centre, cancer care clinics and centres, outpatient clinics, teaching and university hospitals, ER departments	Varied patient population including cancer, gynaecology, neurology, domestic violence, paediatric rheumatology	Mostly doctors and some studies included nurses
LeBlanc et al. (2020) [54]	Scoping review	eHealth technologies used to remotely diagnose and treat patients (including combinations of video-conferences, telephone calls, and remote monitoring devices)	Rural health settings	Not described	Not described
Irvine et al. (2020) [88]	Systematic review	Telephone-delivered interventions	Clinical settings and other settings like educational and occupational contexts	Individuals with clinically diagnosed mental health conditions and those presenting with sub-threshold psychological or emotional difficulties	Not described
Thiyagarajan et al. (2020) [116]	Scoping review	Synchronous videoconferencing for two-way communication	Primary care	Not described	Not described
Gorrie et al. (2021) [70]	Literature review	Telegenetics through videoconferencing	Not described	Patients seen for cancer, prenatal/pediatric genetic counselling	Health professionals utilizing telegenetics
Siegel et al. (2021) [58]	Systematic review	Telephone, Zoom, Epic MyChart video visits	Not described	Not described	Psychiatrists and mental health providers
Dalley et al. (2021) [87]	Systematic review	Telehealth videoconferencing	Primary care (telecardiology and televascular), nursing home, speech language therapy, and postoperative oncology	Not described	Not described
Keenan et al. (2021) [72]	Systematic review	Information Communication Technology (including telehealth and non-telehealth services involving an interaction with the healthcare provider)	Not described	Not described	Not described
Drovandi et al. (2021) [55]	Overview of systematic reviews	Remote monitoring or management technologies including synchronous teleconsultations (phone and video) mobile-based systems for image documentation, mobile phone apps, mobile outreach services, remote review of digital records, home-based monitoring devices	Not described	Adults with or at risk of diabetes-related foot disease	Clinicians managing diabetes-related foot disease
Ferguson et al. (2021) [117]	Systematic review and qualitative meta-synthesis	Wearable technologies or devices that used wearable, continuous and passive monitoring	Range of settings including residential and nursing homes, hospitals, and clinics	Older patients, mean age over 65 years with a heart-related condition	Providers included doctors, nurses, general practitioners
Howard et al. (2021) [94]	Systematic review	Not all studies involved technology-mediated interventions. Types of technology included videoconferencing, telephone and internet-based therapy	Varied settings including inpatient and outpatient settings	Patients with Post Traumatic Stress Disorder related to varied issues including childhood abuse, political violence, war trauma/veterans, illness, complex trauma exposure, dissociative disorders, schizophrenia, sexual assault	Not described
Kinley et al. (2021) [56]	Systematic rapid realist review	Remote consultations including telephone and video consultations	Not described but focus of paper appears to be on primary care	Adults or children diagnosed with asthma	Healthcare providers (professional background not specified)
Sharma et al. (2021) [75]	Unclear (the terms "thematic review" and "literature review" are used)	Telepsychiatry involving videoconsultation	Not described	Not described	Not described
Spelten et al. (2021) [57]	Scoping review of reviews and review of current evidence and guidelines provided by professional bodies	Mostly telephone or videoconferencing, followed by web-based interventions	Not clearly described in all included reviews but some reviews mentioned different settings including hospitals, palliative care, hospices, community primary care	Cancer survivors with different types of cancer including breast (majority), colorectal, prostate, cervical, ovarian	Health care providers including nurses (most frequently), psychologists, physiotherapists, social workers, counsellors, peer workers
Verma et al. (2021) [63]	Rapid review	Telemedicine	Primary care settings only	Not described	Primary care providers (professional background not described)
Wallace et al. (2021) [76]	Scoping	Telehealth	Not described	Patients with different types of musculoskeletal pain at different sites (e.g., spine, upper limbs, lower limbs) and associated with different conditions (e.g., OA knee)	Not described
Wu et al. (2021) [78]	Integrative review	Telemedicine including virtual synchronous visits provided through phone calls or video web conferencing	Not described, but appear to include prenatal care provided in any setting	Pregnant patients (low and high risk)	Nurses, midwives, physicians, or other health care providers providing routine prenatal care, management of gestational diabetes or maternal fetal medicine consultations
de Albornoz et al. (2022) [68]	Systematic review	Telemedicine delivered via telephone or videoconference	Primary care only (including mental health and allied health services)	Adults aged 18 and older receiving primary care, mental health or allied health services, with a range of concerns including acute nonurgent conditions, major diagnoses (including mental disorders), respiratory infections, malnutrition, nicotine dependence, chronic conditions and post-partum care, medically unexplained pain, opioid abuse, cancer	Primary care providers including physicians and allied health staff
Walthall et al. (2022) [77]	Unclear. The term 'Narrative synthesis' is used to describe the methodology as well as the analysis method	Remote consultations including telephone, video, and electronic consultations through text messages, websites or email	Different settings including palliative care, primary care, musculoskeletal care	Not described	Physicians, physiotherapists
Diaz et al. (2022) [69]	Scoping review	Telemedicine including video consultations conducted by Zoom, Skype, FaceTime and via EHR mobile applications	Primary care and specialty clinics	Non-institutionalized, non-chronically ill female adolescents and young adults, ages 10–24 years	Providers serving this patient population
Lampickiene et al. (2022) [73]	Scoping review	Videoconferencing	Not described	Not described	Healthcare professionals including mostly physicians (medical oncology, general practitioners, otolaryngologists, urologists, cardiologists, physiatrists), mental health professionals (therapists and psychotherapists), nurses, advanced practice professionals, dieticians, physical therapists
Lindenfeld et al. (2022) [64]	Scoping review	Synchronous telemedicine consultations through videoconference or audio-only technologies	Primary care settings (large integrated health systems, academic medical centres, veterans affairs clinics)	Not described	Primary care providers (professional background not described)
***Computerized Decision Support Systems***					
Scalia et al. (2019) [118]	Systematic review	Patient Decision Aids designed for collaborative use during clinical encounters	Varied settings including in-hospital and outpatient clinics, hospital units, and primary care	Not described	Providers from varied disciplines including primary care clinicians, specialists, nurses, medical assistants,
Yen et al. (2021) [119]	Systematic review	Patient Decision Aids	Varied settings including clinics, homes and research facilities	Patients on hemodialysis for kidney transplants, cancer screening, cancer surgery, total knee replacement, early intervention for developmental concerns, prenatal genetic testing, chest pain testing	Not described
Čartolovni et al. (2022) [120]	Scoping review	Artificial Intelligence-based medical decision-support tools including machine learning, deep learning, and several papers specifically mentioned IBM's Watson	Not described	Not described	Not described
***Information systems***					
Farnood et al. (2015) [80]	Systematic review	Internet use on smartphones	Primary care settings	Patients over 18 years of age, not described further	Health professionals over 18 years of age, including physicians, nurses, and others
Luo et al. (2022) [85]	Systematic review	Online health information seeking using the Internet	Range of practice settings including primary care clinics, hospitals, and medical specialist practices	Patients with range of conditions including mental health disorders (psychosis, schizophrenia) hematology-related conditions, diabetes mellitus, heart conditions, hepatitis C, dermatological conditions, rheumatological conditions, cancer, multiple sclerosis, reproductive conditions and needs, preoperative consults	Not described
***Multiple technologies across categories***					
Crooks et al. (2009) [121]	Systematic review	Computer use during in-person appointments, Electronic Medical Records	Primary care (family medicine)	Not described	Family doctors
Ludwick et al. (2009) [122]	Systematic review	Computer Physician Order Entry Systems, Electronic Medical Records, Electronic Health Records, Clinical Decision Support Systems and Personal Health Records	Primary care, ambulatory care, community care and acute care settings	Not described	Not described
Kruse et al. (2015) [123]	Systematic review	Web-based portals, medication management tools, mobile monitoring apps connected to portals	Not described, but some references made to hospitals and clinics	Patients with chronic diseases, including diabetes, obesity, heart conditions and cancer	Not described
Barbosa et al. (2016) [61]	Integrative review	Telehealth (telephone and video) and computerized decision support tools	Not described	Not described	Nurses
Crampton et al. (2016) [124]	Scoping review	Health Information Technologies used in face-to-face clinician encounters (desktop, laptop, mobile, tablet)	Primary care (including pediatrics and psychiatry), specialty clinics, internal medicine clinics, hospital departments, home care/OT, inpatient (simulations)	Not described	Not described
Clarke et al. (2016) [125]	Unclear (literature or systematic review)	Internet (including websites, chat rooms, email lists, email with a healthcare provider)	Primary care	Adult patients including women with polycystic ovarian syndrome, patients with hypertension, diabetes, hypercholesterolemia, chronic obstructive pulmonary disease, chronic conditions, non-inflammatory musculoskeletal pain, HPV infection, epilepsy, coronary heart disease, cystic fibrosis, orthopedic conditions, asthma	Not described
Patel et al. (2017) [126]	Systematic review	Computerized systems (mostly EHRs, also included other types like order entry and decision support, or did not specify type of system)	Varied settings including large health systems, regional hospitals, primary care clinics, specialty clinics, surgical clinics, Veterans Affairs settings and simulated laboratory settings	Not described	Not described
Barr et al. (2017) [86]	Systematic review	Information Communication Technologies (including Electronic Health Records, telehealth, online communities and learning resources)	Varied settings including primary care, hospitals, community care, long-term care	Individuals with medically complex conditions, depression, hypertension, obesity, Parkinson’s disease, diabetes, chronic obstructive pulmonary disease, heart failure/arrhythmia, cancer	Family physicians, nurses, nurse practitioners, pharmacists, physical and occupational therapists, speech language therapists, pathologists, dieticians
Rouleau et al. (2017) [53]	Overview of systematic reviews	Information Communication Technologies including management systems (e.g., Electronic Medical Records, Electronic Health Records), communication systems (e.g., email, mobile phone, telemedicine or telehealth using videoconferencing), and computerized decision support systems (e.g., medication management technology)	Varied settings including emergency departments, in-hospital units and clinics, primary care, ambulatory clinics, long-term care, home and community care	Not described	Registered nurses, nurses in training, nursing students
Adjekum et al. (2018) [59]	Scoping review	Digital health	Not described	Patients or the public, not described further	Pharmacists, OTs, PTs, physicians and nurses, medical and nursing students
Palacholla et al. (2019) [127]	Scoping review	Digital Health Technologies (including remote monitoring and management, clinical decision support, patient engagement, televisits, point-of-care, tools providing computer access to clinical data)	Mostly primary care settings	Patients managing hypertension	Not described
Davies et al. (2020) [128]	Systematic review	Web-based mental health therapy (entirely self-directed web-based or blended with face-to-face care)	Hospitals, clinics, general community	Not described	Psychologists, social workers, general practitioners, nurses, psychiatrists
Sunjaya et al. (2020) [84]	Systematic review	Videoconferencing, email, web and application-based platforms and online modules	Not described	Patients with post-traumatic stress disorder	Not described
Hilty et al. (2021) [129]	Literature review	Text, sensors and wearables, e-consultation, store-and-forward technology	Primary care, including referral to and communication with specialists	Not described	Primary care providers and specialists
Noblin et al. (2021) [82]	Systematic review	Patient portal, secure messaging, results reporting, telehomecare, Electronic Health Record, email, online immunization records, electronic Personal Health Record, eRedbook, personal child health record, 2-way messaging with providers	Not described	Adult and child patients, not described	Not described
Al-Naher et al. (2022) [67]	Systematic review	Remote monitoring systems, clinical decision tools, patient health information platforms, online patient self-management tools, educational tools, telephone consultations, peer-support system, pharmacy-based consultation	Patients' homes (majority), community including workplace, hospitals and clinics	Adults diagnosed with chronic heart failure of all severities	Health care professionals involved in their care, professional background not specified
Giordan et al. (2022) [130]	Systematic review	Mobile apps via smartphones or tablets (for telemonitoring with transfer of data to providers, self-management support, patient access to electronic medical records, or direct clinician communication)	Not described	Adult patients with heart failure	Not described
Hartasanchez et al. (2022) [71]	Systematic review	Synchronous (e.g., video consults, technology used in preparation for synchronous consult like symptom tracking device) and asynchronous remote shared decision making (e.g., patient-clinician conversation using apps)	Primary care clinics, tertiary referral centres, and academic departments	Adults with chronic conditions (including cancer, diabetes, vascular or cardiovascular conditions, Parkinson's disease, uterine or prostatic hyperplasia, hip/knee/back pain), young adults with infertility, mental health conditions	Healthcare providers (professional backgrounds not specified)
Shah et al. (2022) [74]	Narrative review	Social media (Facebook, YouTube); mobile apps; websites; text messaging; blended, telephone-based, video-conferencing delivered in real time; multitechnology interventions	Not described	Patients with knee or hip osteoarthritis or pain both pre and post joint replacement surgery	Provider background not specified in some included studies, but mostly included providers from different professional backgrounds like physiotherapists and nurses

### Conceptualization of relationships and trust

Patient-provider relationships were defined in six reviews [83, 85, 91, 110, 121, 125]. One review defined trust in doctor-patient relationships [59]. Provider-provider relationships were not defined and only directly referred to in three reviews [53, 54, 107]. These definitions provided some insight into how authors understood and used the terms ‘trust’ and relationships’ within the context of their review. The reviews also used different terms that were either explicitly connected with relationships or were interpreted by us as related to relationships based on our operational definition. Connections between terms were most often not described and challenging to identify (Table 4).

**Table 4:** Definitions of relationships and trust

Author	Definition
Watkins et al. [110]	Relationships have been described within the context of Relationships-Fit-Visibility Framework i.e., “relationships with health workers and peers as a means of providing support for behavioural change, feedback, and reinforcement”. More generally, they define patient-provider relationships as the patient engaging with the provider.
Crooks et al. [121]	Relationships have been defined in relation to continuity of care which is defined as including three interrelated dimensions (informational, longitudinal/geographical, relational/interpersonal or the development of a trusting relationship between patient and doctor over time).
Clarke et al. [125]	“Relationship-based" care and "therapeutic alliance" are included in the authors' definition of patient-centered care as described below: “The Agency for Healthcare Research and Quality (AHRQ) defines PCC as the relationship-based primary care that meets the individual patient and family’s needs, preferences, and priorities. PCC integrates the disease and illness experience while acknowledging the whole person to create a sharing of power, responsibility, and therapeutic alliance.”
Qudah et al. [83]	The authors use Beach et al.’s four aspects of relationship-centred care and reconstruct descriptors of each aspect specific to mHealth: 1.Relationships in healthcare ought to include dimensions of personhood as well as roles 2.Emotion and empathy are important components of relationships in healthcare 3.All healthcare relationships occur in the context of reciprocal influence 4.Relationship-centred care has a moral foundation
Rathert et al. [91]	Fostering healing relationships is defined as “characterized by trust and rapport. Everyone should understand each other’s roles. Providers should take the lead in addressing issues that might prevent patients and families form being actively involved. A trusting relationship can depend on and facilitate communication”.
Luo et al. [85]	The term “physician–patient relationship” is used and described as “second only to that of family”. The “traditional physician–patient relationship” is described as one where “physicians made decisions and patients obeyed them”, which is now transitioning to one of “mutual participation, shared power and responsibility”. Quality of communication is seen as affecting the physician–patient relationship.
Adjekum et al. [59]	"Trust is oftentimes illustrated as a relationship between one party (a trustor) and another (a trustee) with optimistic anticipation that the trustee will fulfill the trustor’s expectations."
	"Whether or not it is appropriate to talk about trust between people and inanimate objects—such as technological products—remains an open question in the literature."
	'Trust enablers' refer to those factors that encourage stakeholders' trust in digital health 'Trust impediments' denote the factors that can potentially hinder trust

**Fig. 3:**
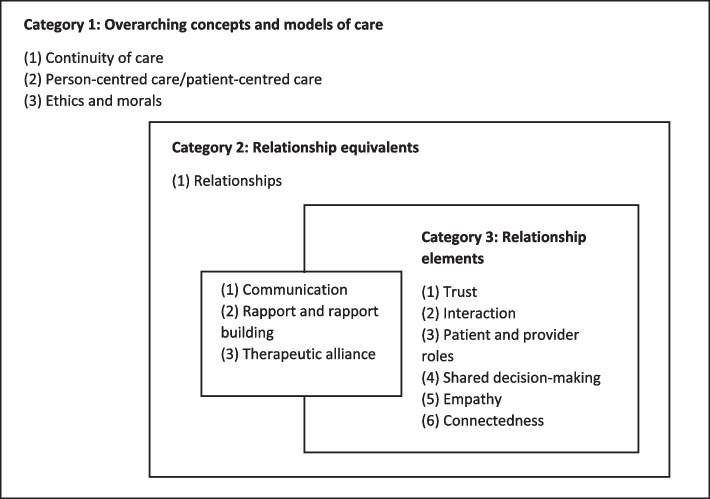
Terms used to describe patient-provider relationships

The terms used to refer to patient-provider relationships were organized into three non-mutually exclusive sets: 1) Overarching concepts and care models; 2) Relationship equivalents or elements; and 3) Relationship elements. Overarching concepts and care models (category 1) included terms that encompassed relationships, such as continuity of care, person-centred/patient-centred care, ethics, and morals. Relationship equivalents included terms that were used interchangeably with relationships, whereas relationship elements included terms that were encompassed within relationships. Terms that were both relationship equivalents and elements (category 2) included communication, rapport/rapport-building, and therapeutic alliance. Terms that were only relationship elements (category 3) included trust, interaction, patient and provider roles, shared decision-making, empathy, and connectedness. Some terms that came up less frequently and consistently (e.g., information sharing, support, collaborative care) could not be meaningfully mapped and connected to other concepts. Future research could explore the interpretation and use of these other less frequently used terms. Figure 3 indicates our interpretation of the connections between different terms used for patient-provider relationships.

With respect to provider-provider relationships, we were unable to categorize terms as the smaller number of reviews made it challenging to identify patterns and connections between terms. Terms mostly appeared to be used either interchangeably with relationships or as standalone terms and included: communication, collaboration, interaction, information sharing/exchange, connection, support.

### Impact of eHealth on patient-provider relationships

Forty-seven reviews reported a mix of positive and negative impacts of eHealth on patient-provider relationships [16, 54, 57, 59, 62, 64–68, 70, 72, 74, 73, 75, 76, 78–81, 85, 88, 89, 91, 92, 95–99, 104–108, 112, 113, 116, 117, 120, 122–124, 126–129] (e.g., communicating via technology created a distance between the patient and provider in some instances, but also reduced loneliness in others). Nineteen reviews reported mainly positive impacts (e.g., more collaboration and closeness between patient and provider) [53, 55, 56, 60, 71, 77, 83, 100, 102, 103, 109–111, 115, 118, 119, 121, 125, 130] while seven reviews reported mainly negative impacts (e.g., reduced conversation flow) [58, 61, 63, 69, 82, 87, 114]. Three reviews noted no impact of technology on patient-provider relationships [84, 93, 94]. We also noted a collection of factors that influenced whether the impact of eHealth on patient-provider relationships and trust was positive, negative, or neutral. We categorized the influencing factors as patient-related, provider-related, technology-related, and organizational factors. Each category is described below with examples from relevant reviews. Table 5 displays the frequency of factors across different types of technology. Additional file 2 describes the factors and impact reported in each study discussing patient-provider relationships.

**Table 5:** Factors influencing the impact of technology on patient-provider relationships

	**Management systems**	**Communication systems**	**Computerised decision support systems**	**Information systems**	**Multiple technologies**
***Patient-related factors***					
Patient perceptions, expectations, motives, and concerns	****	**********		**	**
Patient functional ability		******			
Patient communication skills and participation		*			
Sociodemographic factors		********	*		*
Familiarity, consistency within relationship or presence of pre-existing relationship		*****			
***Provider-related factors***					
Provider communication skills and technology use style	*******	******	*		****
Provider qualifications/level of experience	*	*			
Provider perceptions, reactions, and attitudes	***	**********		**	**
***Technology-related factors***					
Type of care delivery modality		************			***
Technology design and features	*****	****	*		*****
***Other (institution or organizational factors)***					***

Asterisks [*] used indicate the number of reviews discussing each category of technology (i.e., management systems, communication systems etc.)

#### Patient-related factors

1. *Patient perceptions, expectations, motives, and concerns*were the most reported factors influencing relationships and trust (18 reviews), particularly in reviews focusing on management and communication systems. For example, patients had greater trust in providers and satisfaction with the relationship when using Electronic Health Records (EHRs) and telemedicine when they perceived providers as competent, knowledgeable, or experienced [96, 113]. Patient perceptions that remote patient monitoring would replace personal care was related to a negative impact on communication, interaction, and trust, whereas feeling like an “equal partner” when providers included them in discussions about their data was related to a positive impact on relationships [112].
  This﻿ factor was also discussed in two reviews focusing on information systems. For example, a positive impact on relationships was noted when patients’ motives for seeking online health information were to support rather than challenge the therapeutic relationship [80] and when they were willing to discuss online health information with the provider as compared to when they were afraid of challenging the provider’s authority [85].
2. *Patient functional ability* was linked to patient-provider relationships in six reviews mostly discussing communication systems. For example, the alliance built through videoconferencing was seen as impaired for patients with epilepsy, post-traumatic stress disorder [62] and cognitive-behavioural challenges [58]. Communication challenges during teleconsultations were reported with patients with visual and hearing impairments [63, 64]. With mental health, the patient-provider relationship was sometimes seen as better (patients were more willing to share information virtually) and sometimes worse (providers perceived a need for human contact to facilitate recovery) when using virtual modalities [54]. One review noted that patients and providers felt that the ability of remote consultations to facilitate patient empowerment and participation could change as the patient’s illness progressed [77].
3. *Sociodemographic factors* were related to relationships and trust in 10 reviews, mostly focusing on communication systems. With respect to *age,* remote consultations and telehealth were linked to the development and sustenance of positive and trusting relationships particularly in younger [56] and more computer literate patients [57]. Contrastingly, one review noted that older patients felt that telehealth facilitated discussions with their provider and supplemented standard visits [55].
  Three reviews indicated that *language barriers* can lead to communication systems having a negative impact on patient-provider communication [63–65] and one noted that language barriers were more common with patients in high social vulnerability areas [63].
  Two reviews indicated that the impact of communication systems like telemental health and mHealth on patient-provider relationships and trust can vary according to *socioeconomic status* [16, 58]. Two reviews discussing management systems [59] and multiple types of technology [60] reported socioeconomic status as a factor or barrier influencing trust and *relationships*.
  Two reviews discussed the impact of communication and management systems on relationships and trust in *minority/disadvantaged groups*. One noted a negative impact on relationship-building during telephone consultations for minority patients [57]. The other reported a positive impact on patient trust in providers for disadvantaged patient groups related to the use of Patient Accessible Electronic Health Records (PAEHRs) [66].
  Table 6 outlines the varying impact of eHealth by functional ability and sociodemographic factors.

**Table 6:** Impact of eHealth in different groups

**Does the impact of eHealth on relationships and/or trust differ by age group?**	**Type of technology**
LeBlanc et al. [54] – Increased willingness and comfort with sharing information virtually particularly in teenagers.	Communication systems
Drovandi et al. [55] – Older patients particularly felt that telehealth facilitated discussions with providers and supplemented standard visits.	Communication systems
Kinley et al. [56] – Remote consultations lead to creation and sustenance of positive working relationships, particularly in younger patients.	Communication systems
Spelten et al. [57] – Young computer literate people reported being able to better develop a trusting relationship with their provider via telehealth compared to older people.	Communication systems
**Does the impact of eHealth on relationships and/or trust differ by socioeconomic status?**	**Type of technology**
Siegel et al. [58] – providers felt that telephone use is preferred by patients of lower socioeconomic status, but is less personal and creates more challenges in collecting information and maintain therapeutic alliance without access to facial and body cues.	Communication systems
Odendaal et al. [16] – mHealth led to new forms of engagement and relationships with clients and communities, with some providers expressing concerns about increased inequity from using expensive equipment and others believing that access to mobile devices was beneficial to clients and communities who could not afford one.	Communication systems
Adjekum et al. [59] – Socioeconomic status is noted as a personal factor influencing trust, but not elaborated on. They conclude that more research is required.	Multiple types of technology
Wark et al. [60] – Low health literacy and low socioeconomic status are noted to be barriers to integrating Social Determinants of Health data into EHR specifically with respect to patient-provider relationships, but not explained further.	Management systems
**Does the impact of eHealth on relationships and/or trust differ according to the patient’s functional ability?**	
Barbosa et al. [61] – Clinical condition mentioned as a potential communication barrier in telehealth context, but not explained.	Communication systems
Simpson et al. [62] – Providers feel that therapeutic alliance is impaired in patients with epilepsy and PTSD. The review also notes that videoconferencing might be more appropriate for some types of patients with certain types of mental health challenges who have a “heightened need for distance and safety” whereas paranoid and avoidant personality characteristics or difficulty trusting others may limit effectiveness of videoconferencing.	Communication systems
LeBlanc et al. [54] – Patients were more willing to and comfortable with sharing information about mental health concerns virtually, but providers perceive need for human contact.	Communication systems
Siegel et al. [58] – Providers felt that remote delivery creates challenges in focusing in the presence of interruptions and distractions, particularly for patients with cognitive behavioural challenges.	Communication systems
Verma et al. [63] – Providers reported communication challenges during telemedicine, particularly for patients with hearing impairments.	Communication systems
Lindenfeld et al. [64] – Telemedicine can potentially decrease “human connection”, make it challenging to convey empathy, and create communication barriers for patients with visual and auditive impairments.	Communication systems
**Does the impact of eHealth on relationships and/or trust differ according to language?**	
Henry et al. [65] – communication challenges during videoconferencing can be exacerbated by language barriers.	Communication systems
Verma et al. [63] – providers noted communication challenges during telemedicine when there were language barriers, which were more commonly noted with patients in high social vulnerability index areas.	Communication systems
Lindenfeld et al. [64]—Telemedicine can potentially decrease “human connection”, make communication and conveying empathy challenging with patients speaking non-native languages.	Communication systems
**Does the impact of eHealth on relationships and/or trust differ for patients belonging to minority/disadvantaged groups?**	
Spelten et al. [57] – Patients and providers perceived limited access to non-verbal cues and capacity for relationship building via phone, particularly for minority participants.	Communication systems
Benjamins et al. [66] – Disadvantaged groups (referring to ethnic minorities and those with lower educational levels) experience increased trust in White providers through increased access to their records and transparency and are likely to benefit more.	Management systems
**Does the impact of eHealth on relationships and/or trust differ according to gender?**	
Barbosa et al. [61] – Gender is mentioned as a potential communication barrier in telehealth context but not explained further.	Communication systems
4. *Familiarity and consistency within the relationship or presence of a pre-existing relationship*was reported in reviews discussing communication systems (five reviews). For instance, regular and effective patient-provider communication was noted when the provider remained the same [117]. Patients were found to report mostly positive experiences when telehealth facilitated maintenance of a pre-existing relationship [57]. A pre-existing patient-provider relationship when using remote consultations was linked to positive outcomes including enabling providers to engage patients in shared decision-making and self-management [56] and better treatment continuity and clinician outcomes [68]. However, Verma et al. reported that patients found telemedicine impersonal even when they knew their provider [63].

#### Provider-related factors

1. *Providers’ communication skills and technology use style* (i.e., provider’s style of using technology during an in-person visit or for remote patient communication) were frequently connected to the impact of technology on relationships and trust (18 reviews), particularly in reviews discussing management and communication systems.
  With use of management systems like EHRs during in-person visits, examples of provider behaviours that impacted relationships positively included making computer use less obvious; inviting patients to look at the screen to facilitate conversation particularly during sensitive discussions; maintaining eye contact and conversation with patients [79, 122, 126]; giving patients time to reflect by turning away to enter data on the computer [122]; using technology as a discussion tool for emotional support [69] and collaborative planning and documentation [104]. On the other hand, screen gaze [79, 89, 91, 92], keyboarding [79, 89, 91], closed body posture [79], and indirect facial orientation [91] had a negative impact.
  With respect to communication systems (teleconsultations and remote monitoring systems), providers’ ability to develop a “video presence” [65], adjust communication style by using non-verbal cues [62, 65], provide undivided attention and create a supportive and relaxed environment [77], use technology for direct and indirect patient communication [110] and information exchange by sharing charts and test results [113] were linked with a positive impact on relationships.
  Nine reviews suggested that the negative impact resulting from the provider’s technology use style and communication can be mitigated by: using strategies specific to the care delivery modality (telephone or video consultation) [58, 64, 77, 124, 129]; provider training in technology use [89, 91], in the limitations and regulations related to technology and in judging appropriateness of the modality [107]; considering the context and patient preferences and experiences while designing and implementing new technologies [89, 72]; and setting clear expectations between patient and provider [81].
2. *Provider perceptions, reactions and attitudes* were reported in 16 reviews, mostly those discussing communication systems. For example, the impact of mHealth could be positive or negative depending on provider perceptions about the need for face-to-face contact (some wanted in-person contact or expressed concerns with “impersonalization” of interactions), access (some perceived increased access to services through mHealth), and the need for boundary setting (some felt the need to set boundaries to being contactable outside working hours) [16].
  Provider perceptions and beliefs were also noted in reviews discussing other types of technology. For example, negative provider perceptions and concerns around the potential for management systems like EHRs to reduce time spent with patients and interfere with direct care provision was linked to a negative impact on relationships [97]. Relating to information systems, a positive impact of patient online health information seeking was noted when providers believed that patients have the right to be informed and created an open environment, whereas a negative impact resulted when providers believed that patients seek online information because they don’t trust them [80].
  Differences in impact were also found depending on whether a provider had used technology or not. For instance, providers using management systems (EHRs) and communication systems (remote monitoring equipment and videophone) generally perceived greater positive impact compared to nonusers who anticipated challenges [98, 106]. Two reviews noted that providers’ initial concerns about potential negative impacts of teleconsultations changed to a perceived positive impact after use [75, 77].
  In one review, provider perceptions of patient expectations influenced the impact on relationships and trust. For example, providers believed that patients preferred in-person interactions and that use of patient-generated health data would exacerbate social isolation and hinder collaboration [81]. Provider and patient perceptions sometimes conflicted. For example, providers felt that patients found technology difficult to use; however, patients felt that technology reduced anxiety and improved self-management [106].

#### Technology-related factors

1. *Type of care delivery modality* (video, phone, or in-person) was the most reported technology-related factor (15 reviews) discussed in reviews of communication systems.
  In-person vs. remote (phone and video) consultations
  Two reviews found that the therapeutic alliance did not differ for remote and in-person interventions [93, 94] while one found that it was stronger over teleconsultation compared to in-person [68]. Patients and providers reportedly perceived that remote consultations build trust [129], facilitate strong alliances and quick exchanges over time [129], continuity and consistent access to the same provider [56, 129], individualized and timely support [56], leading to positive working relationships [56]. In contrast, one review noted that in-person visits allowed for providing richer information and advice compared to teleconsultations [68] and another reported that increased trust created through asynchronous communication could lead to assumptions about other users’ intentions (e.g., assumption that the other user is being truthful) [129].
  One review reported varying perceptions of virtual visits, with some patients and providers noting greater family inclusion and support and others perceiving less compassion, empathy, and discomfort with the possibility of multiple people watching during video visits [78]. Another noted that providers perceived blended care (mix of in-person and remote care) as “different” but “not necessarily worse” than in-person care; some providers were surprised by their ability to build relationships online and found that blended models provided more opportunities for rapport, support, and monitoring [128].
  In-person vs. phone consultations
  The alliance over phone consultations was found to be “different” compared to in-person care in one review focused on psychological therapy; greater task/treatment focus over the phone appeared to compensate for a reduction in bond, made it easier to stick to time boundaries, and, in one review, patients found the visual anonymity beneficial [88].
  In-person vs. video consultations
  Compared to in-person consultations, relationship-building over videoconferencing took longer and resulted in reduced conversation flow [111]. The therapeutic alliance could either be equivalent, improved, or impaired in videoconferencing compared to in-person depending on the patient’s diagnosis and the therapist’s and patient’s ability to adjust communication styles [62]. Providers found that videoconferencing provided more time to deliver personalized care and patients perceived more individual attention and focus via videoconferencing compared to in-person consultations after initial scepticism [76]. Videoconferencing was also reported to lead to loss of professional boundaries when patients were unintentionally able to view providers’ homes, leading to patients getting more personal information than the provider would like [58].
  Phone vs. video consultations
  Compared to phone consultations, patients and providers perceived that videoconferencing increased closeness, engagement, and continuity [111], facilitated rapport building [68] and non-verbal communication [57]. Phone consultations reportedly limited capacity for relationship-building and maintaining therapeutic alliance due to limited access to non-verbal cues [57, 58, 68, 78], particularly among minority participants [57]. Some patients desired to see the provider’s reaction and perceived inadequate time for questions during audio-only visits as compared to video and in-person consultations [78]. However, some also valued the “undivided communication” offered via phone-based interventions [74]. One review noted that patients reported more positive experiences with both phone and video consultations being used together [57].
2. *Technology design and features*were reported in 10 reviews, across management, communication, and information systems. For example, personalized design, real-time monitoring, and two-way communication through mHealth apps were reported to improve information sharing and continuity of care, facilitate power and responsibility sharing, and increase trust [83]. Features like provider access to trends and summary measures [81], joint viewing of imaging results with patients [90], screensharing and document editing [56], and integration of social determinants of health [60] in EHRs and Patient Generated Health Data (PGHD) supported collaboration, communication, and shared decision-making. Technology that provided opportunities for communication was perceived by patients to reduce isolation, increase trust in the provider, and led to providers perceiving patients to be “more open”, whereas technology that reduced communication led to patients missing human contact and created a “distance” [67]. One review identified usability (e.g., ease of use) as important for synchronous technology like video consults and asynchronous remote decision-making technology to facilitate partnerships and interactions [71].

#### Organizational factors

Organizational factors relating to implementation and use of technology were reported in three reviews that discussed multiple types of technology. For example, implementers were noted to be concerned about the potential negative impact of technology like Electronic Medical Records (EMRs), EHRs, and computerized clinical decision support systems on patient-provider relationships [122]. The absence of guidelines and insufficient training for using technology were reported as impediments, and stakeholder engagement as an enabler of stakeholder trust in technology [59]. Synchronous technology like video consults and asynchronous shared decision-making technology could reportedly facilitate “partnerships” and “remote interactions” if factors like training in technology use and broadband access were addressed [71].

### Impact of eHealth on provider-provider relationships

eHealth appeared to have a positive (7 reviews) [53, 55, 98, 102, 111, 113, 114], negative (6 reviews) [58, 64, 73, 86, 97, 101], or mixed (9 reviews) [16, 54, 67, 77, 87, 90, 99, 107, 108] impact on provider-provider relationships depending on provider-related, technology-related, and organizational factors. Examples from relevant reviews describing each category of factors are discussed in this section. Table 7 displays the frequency of each factor across types of technology. Additional file 3 describes the factors and impact reported in each study discussing provider-provider relationships.

**Table 7:** Factors influencing the impact of technology on provider-provider relationships

	**Management systems**	**Communication systems**	**Multiple technologies**
***Provider-related factors***			
Provider communication and technology use skill and style	**	***	
Provider attitudes towards and perceptions of technology	*		*
***Technology-related factors***			
Technology features and design	*	*	*
Task-technology fit		*	
***Other (institution or organizational factors)***	*	*	

Asterisks [*] used indicate the number of reviews discussing each category of technology (i.e., management systems, communication systems etc.)

#### Provider-related factors

1. *Provider communication and technology use skills/style* were reported to influence provider-provider relationships in four reviews discussing management and communication systems. With respect to communication systems, a negative impact was noted when providers had impaired technical communication skills like sending delayed email responses (potentially leading to friction) and because of limited non-verbal cues and informal contact in virtual teams (leading to weaker working relationships) [107]. On the other hand, clarification actions (or “utterances” intended to clarify and understand) between providers while using videoconferencing equipment were reported to enhance collaborative working [87].
  For management systems, providers with higher skill in technology use perceived greater benefit from EMRs [98]. Providers’ technology use style (e.g., frequent use of the copy-and-paste function) led to “cluttered” notes and limited providers’ ability to develop “shared understandings” [101].
2. *Provider attitudes towards and perceptions of technology* were noted to impact team relationships in two reviews (one discussing mana>gement systems and the other discussing multiple technologies). For example, negative provider perceptions of EMR as “management control systems” were reported to infringe on privacy and autonomy [97]. Providers’ lack of willingness to learn how to use online communities was reported to be a barrier to the otherwise positive impact of the technology on interprofessional collaboration [86].

#### Technology-related factors

1. *Technology features and design* were linked to a negative impact on team relationships in three reviews (one discussing management systems, one discussing communication systems and the other discussing multiple types of technology). Relating to management systems, the templated structure of EHR, lack of ease in informational retrieval, lack of representational structures for communicating nurse, patient, and psychosocial perspectives on care had a negative impact on team communication [101]. With communication systems, unidirectional paging systems were noted to impair communication [90]. One review discussing multiple types of technology reported positive or negative provider perceptions of team communication and teamwork depending on the ability of the technology to connect members (e.g., when technology did not have features that allowed physicians to connect with specialists, it negatively impacted communication) [67].
2. *Fit between task and technology* was reported in one review discussing multiple types of technology; selecting communication technology that fits the task was found necessary to support team routines and communication [107].

#### Organizational factors

Availability of resources like standards and guidelines, training, strategic and creative adaptations was reported to be vital for facilitating virtual team operations and dynamics [107]. The extent of perceived benefit of EMR was linked to the size of the practice, such that larger practices saw greater benefit of EMR in communicating with other providers and organizations.

## Discussion

This review of reviews intended to better understand how eHealth impacts patient-provider and provider-provider relationships and trust in primary care by examining existing evidence syntheses. We found 79 reviews that described the impact of management systems, communication systems, information systems, and computerized decision support systems on relationships and trust. Most of the reviews discussed patient-provider relationships and only a small number focused on provider-provider relationships. Overall, management and communication systems were the most frequently discussed types of eHealth technologies and they appeared to have a mixed impact (both positive and negative) on patient-provider and provider-provider relationships and trust.

A steady increase was observed in the number of reviews emerging in this area, particularly in 2021 and 2022. However, only a few intentionally examined and clearly defined relationships and trust. Most of the included reviews had explored the impact of eHealth on relationships as part of another primary aim. Therefore, this impact and the influencing factors were not always explicitly or directly described. This made it challenging to understand what impact the use of technology was having on relationships and why, and often called for us to make connections based on our interpretations. The fluid and expanding nature of eHealth as a group of technologies [14] further adds to the complexity of this issue. For the sake of convenience, we limited our analysis to the four types of eHealth technologies within Mair et al.’s classification [15].

The terms ‘relationships’ and ‘trust’ were not defined in most of the included reviews and several interrelated terms such as ‘communication’ and ‘information-sharing’ were used without drawing out clear connections between each other. Often there appeared to be an underlying assumption that the reader would share the same implicit definition as the authors. Additionally, limited reporting of the authors’ epistemological background made it difficult to unpack these concepts in a meaningful manner. This resulted in a definitional soup or lack of conceptual clarity on what ‘relationships’ and ‘trust’ mean within the context of a specific review. Our analytical challenges in disentangling and interpreting the various terms used made it difficult to determine the impact of eHealth on the different elements or aspects of relationships. This finding points towards the need for better taxonomies in this area that conceptualise relationships, trust, and interrelated terms within the context of eHealth. The conceptualisation we have proposed in this review (Fig. 3) could serve as a starting point that could be built on using participatory approaches with experts (e.g., patients, caregivers, providers, managers) such as Delphi or deliberative methods [131].

Our analysis revealed a mixed impact of eHealth on patient-provider relationships and trust. This impact appeared to be positive, negative, or mixed depending on different influencing factors (patient-, provider-, technology-related, and organizational factors or a combination of these). These influencing factors were not always mentioned directly (if mentioned at all) in the included reviews and were often difficult to identify, possibly indicating the need for more work that is directly focused on understanding how these human and non-human factors might be impacting relationships and trust while using technology.

Of the patient-related factors, *‘patient perceptions, expectations, motives, and concerns’* were most frequently found to influence the impact of management and communication systems on patient-provider relationships. Patients often seemed to perceive a positive impact of these types of technology on the relationship when they perceived that it supported personalised and collaborative care. Another patient- and provider-related factor that came up in more recent reviews (from 2021 onwards) and was associated with a positive impact on the patient-provider relationship was *familiarity or presence of a pre-established relationship*prior to using communication systems like telehealth. These findings suggest that these types of technology are more likely to positively impact relationships and trust when used as part of hybrid care delivery models (where virtual care is used to support patient-provider relationships that have been established through initial in-person interactions) rather than a “digital-first” approach [13]. Similar recommendations have been provided in recent reports and policy documents to guide the use of technology in primary care delivery. For instance, the 2022 Virtual Care Task Force Report in Canada notes that this type of care may be better used “in the context of an ongoing relationship with a family physician or specialist and their care team” ([10] p17). Likewise, the American College of Physicians Policy Recommendations on telemedicine recommend that it “can be most efficient and beneficial between a patient and physician with an established ongoing relationship” ([132] p788).

Our analysis found a small number of reviews that discussed the impact of eHealth on patient-provider relationships (and none on provider-provider relationships) using an equity lens. Equity and the differential impact of technology among different groups on relationships was not considered as a primary aim of most reviews and usually reported as part of other findings, suggesting a need for a more explicit focus on this aspect in future studies. We found a possible differential impact of communication systems (and less frequently of management systems) on patient-provider relationships based on certain *sociodemographic factors*. eHealth mostly appeared to positively impact patient-provider relationships among younger patients, but there was some evidence that this positive impact could extend to older patients as well. These findings are similar to Rodgers et al.’s review [50] that found that although younger healthier patients tend to use digital consultations more, some older patients do use it as well. The impact of eHealth was also linked to the patient’s *functional abilities and/or health condition*. When there were language barriers between patients and providers and for patients with visual, auditory, and cognitive-behavioural challenges, eHealth appeared to negatively impact relationship. In the case of mental health conditions, a varied impact was reported. Therefore, eHealth needs to be used judiciously in these situations, possibly by identifying ways to work through challenges that may arise while working with some patients (for example, by offering patients a choice between virtual and in-person consultations, using virtual consultations as a supplement to in-person care only when preferred or needed, designing technology that better fits individual patients’ needs). Overall, these findings indicate that it is important for providers and organizations to be mindful of these sociodemographic factors and patient preferences in order to facilitate relationship building and maintenance when implementing eHealth solutions. Providers and organizations also need to consider existing inequities in terms of digital literacy and patient access to technology and internet connectivity to ensure that the use of eHealth does not exacerbate existing healthcare disparities [133]. Designing and adapting technology that meets the needs of different patient groups can also ensure that the positive impacts of technology on building relationships and trust with these groups are not lost.

Among the provider-related factors, *‘provider communication skills and technology use style’*(in relation to management and communication systems) were the most frequently reported, particularly during teleconsultations as well as relating to the use of EHRs during in-person consultations. When providers were able to successfully use technology-specific communication skills (like effective non-verbal communication during remote consultations and while accessing EHRs during in-person consultations), there was a positive impact on relationships and trust. While there is already evidence to suggest that provider communication and interaction styles can influence the therapeutic alliance [134], our findings add to this by highlighting the need for providers to adapt these communication skills to the type of technology being used in order to effectively build relationships with patients. While previous research has highlighted the need to train providers in communication and technology use [49], our review specifically brings out the possible benefits of training on optimizing the positive impact of technology on the patient-provider relationship and trust, and how this training may need to account for patient characteristics and needs, technology functionality and organizational contexts. Initiating training early on during medical school and offering continued opportunities for training during post graduate education and through continuing professional development can help providers build skills in using and communicating via technology.

*‘Provider perceptions, attitudes, and concerns’*(in relation to communication systems) were also frequently found to influence the impact of eHealth on patient-provider relationships and trust. Although negative provider perceptions about technology sometimes seemed to have a negative impact on the patient-provider relationship [80, 97], we found that these perceptions could change after providers use technology (see for example Walthall et al., [77] Bassi et al., [98] Brewster et al., [106] and Sharma et al. [75]). We also found that there were some discrepancies between providers’ perceptions of patient expectations and patients’ actual expectations regarding technology use (see for example Brewster et al. [106]). These findings could be because included reviews sometimes appeared to report providers’ perceptions of technology based on its anticipated rather than experienced impact on relationships and trust. It was often challenging to distinguish which of the two the review focused on and making this distinction may have helped us analyse the findings better. More research that collects patients’ and providers’ actual experiences of using technology and its impact on their relationships could help better understand the experienced rather than perceived impact. As well, mutual clarification of expectations regarding use of technology between patients and providers can help optimize its positive impact on their relationship with each other.

With respect to technology-related factors, the *type of care delivery modality* was most frequently found to influence the impact of communication systems on patient-provider relationships. We found mixed evidence on the impact of different types of care delivery modalities (phone, video and in-person consultations). While describing the impact of communication systems on relationships, some reviews did not distinguish between telephone and video consultations when referring to virtual care (see for example, Keenan et al. [72]). As a result, it was difficult to determine which care delivery modality had positive or negative impacts and when. *Technology design and features* were also found to influence the impact of management, communication, and information systems on patient-provider relationships, with a more positive impact noted with technology that facilitated collaboration and communication. These technology-related factors were often reported along with patient- and provider-related factors. For example, what was considered an appropriate care delivery modality depended on patient and provider perceptions (such as in Penny et al. [111] where providers perceived that videoconferencing prolonged the relationship-building process compared to in-person consultations). This suggests that considering these technology-related factors together with person-related factors and targeting the modifiable factors (e.g., increasing awareness and education to change patient and provider perceptions and attitudes towards technology, training providers in communication skills, and designing and choosing technology that meets patient needs) can help achieve good technology-person fit to help facilitate positive patient-provider relationships. Notably, some common technology-related measures like satisfaction were not represented in these reviews, suggesting a potential gap in understanding how usability measures like satisfaction may play a role in patient-provider and provider-provider relationships [135].

Given the very small number of reviews that discussed the impact of eHealth on provider-provider relationships, we were unable to clearly determine the impact by the type of technology. However, the influencing factors that our analysis identified were similar to those influencing the impact of patient-provider relationships. Impaired provider communication and technology use style (such as poor email communication skills and ineffective use of EMR functions), negative provider perceptions of technology, unwillingness of providers to learn about technology, and technology design that did not facilitate communication or ease of use were linked with a negative impact on provider-provider relationships. Organizations can potentially address these factors through strategies such as encouraging initial in-person communication and frequent and continuous communication between providers [136], improving providers’ knowledge of and motivation to use technology [136], and choosing technology that fits with team members and the situation [137]. As teams increasingly work in hybrid environments, organizational behaviour literature can provide valuable insights into optimal ways in which teams can build relationships [138].

Although some of the reviews included in our study provided a few recommendations for the use of technology in primary care settings, these were not always clearly stated or presented as actionable strategies, nor did they directly focus on relationships or trust. Our review addresses this gap by presenting some key recommendations and implications for different stakeholders (such as patients, providers, managers, policy makers, educators, and technology developers) relating to optimal ways to design and use eHealth to facilitate relationship and trust building in different aspects of primary care (such as care delivery, care coordination, team communication, and training/education). These recommendations have been proposed based on the authors’ analysis of the findings from the included reviews and are outlined in Table 8.

**Table 8:** Recommendations for integrating eHealth in primary care to facilitate relationship and trust building and maintenance

1. While determining the appropriate type of technology to be used in a specific situation, assess the situation and the people involved (patients and providers) to determine their perceptions, expectations, concerns, attitudes, and motives relating to technology.
2. Discuss perceptions, expectations, and concerns of all the involved stakeholders openly and clarify any assumptions or misconceptions that stakeholders may have about each other, specifically relating to how they would like technology to be used (or not) in their care.
3. Consider technology-related (e.g., type of care delivery modality, technology design) and person-related factors (e.g., patient and provider perceptions about technology) in tandem during technology design and implementation to achieve good person-technology fit.
4. Consider the use of communication systems like telehealth when the patient and provider have a pre-established relationship.
5. Train providers in using technology, developing technology-specific communication skills, and adapting existing communications skills for technology-mediated interactions to facilitate relationship and trust building with patients and other team members.
6. Consider how technology may likely impact relationship and trust-building differently among different sociodemographic patient groups during technology design and implementation. Use communication systems like telehealth judiciously and be mindful of patient preferences for technology use, particularly in the case of language barriers and with patients with visual, auditory, and cognitive-behavioural challenges.

## Strengths and limitations

By focusing on the relational aspects of primary care in the context of eHealth technologies, this review of reviews addresses an important issue, particularly in the current post-pandemic context where primary care settings are increasingly contemplating how best to integrate technology into care delivery. The recommendations offered for different stakeholders within primary care can inform decision-making around when and how to use different types of eHealth technologies. The search strategy for this review was rigorously developed and implemented. Although single reviewer screening may have led to some relevant articles being excluded, we attempted to minimize this by conducting multiple rounds of agreement checks and discussions between team members to ensure consistency during screening and data extraction. A quality appraisal of each included review was not indicated as this review aimed to provide an overview of existing knowledge in the area [139]. This may have also contributed to our including a wide range of literature thereby providing a comprehensive synthesis of the evidence in this area. The findings of this review also need to be considered in light of certain limitations. Firstly, as relationships and trust were discussed using several interrelated terms that were not always clearly defined, our analysis and findings are based on our interpretation of these terms. We acknowledge that these terms could be interpreted in multiple ways and that the authors of the included reviews may have their own interpretations. The conceptualization presented in this paper represents one way of interpreting these terms. This variation in terminology used and interpretations could have also led to some relevant articles being excluded.

As this study focused on reviews rather than studies discussing individual technologies, the type of technology discussed in different reviews had to be abstracted to high-level categories using an existing classification system (communication, management, information, and computerized decision support systems). As a result, it was difficult to determine the type of impact (positive, negative, or neutral) of individual technologies. Most of the included reviews discussed communication and management systems. As very few reviews discussed computerized decision support systems and information systems or discussed these along with other types of eHealth technologies, it was hard to draw meaningful conclusions about these two types of technologies. While beyond the scope of our study, we do recognize that patient and provider relationships in primary care settings may be influenced by access to and care delivery from other care providers and specialists which is not captured in our results. The findings presented are mostly reflective of the impact of communication and management systems on relationships and trust in primary care settings and should be considered within this context.

## Conclusion

eHealth impacts relationships and trust in positive and negative ways depending on how it is used and who is using it. The potential positive impacts can be lost if it is not used effectively, and negative impacts can be mitigated or compensated for through different strategies, such as designing and using technology that meets the needs of the situation and people involved, and training providers in using and communicating appropriately with technology. The findings of this review have implications for healthcare providers, patients, managers, educators, policy makers, technology developers, and other stakeholders’ decision-making around optimal ways to integrate eHealth in primary care to facilitate relationship-building and maintenance.

### Supplementary Information

**Additional file 1. **Search strategy.

**Additional file 2. **Impact of technology on patient-provider relationships.

**Additional file 3. **Impact of technology on provider-provider relationships.

## Data Availability

Data generated and analysed during this study are largely included in this published article (and its supplementary information files). Raw data sets used to initially collect and sort data can be made available upon request.
