# Supplementary material for: The impact of eHealth on relationships and trust in primary care: a review of reviews
Source: BMC Prim Care. 2023 Nov 3;24:228. doi: 10.1186/s12875-023-02176-5 (PMC10623772; doi:10.1186/s12875-023-02176-5)
Supplement: Supplementary file 1 — Additional file 1. Search strategy. [file 12875_2023_2176_MOESM1_ESM.docx]

**Additional file 1: Search strategy**

**Ovid MEDLINE Search**

1. exp professional-patient relations/ or exp physician-patient relations/
2. exp Interprofessional Relations/
3. patient care team/ or nursing, team/
4. Trust/
5. ((Patient* or client*) adj3 (provider* or physician* or clinician* or professional* or health professional*)).tw,ab,kf.
6. ("doctor-patient relation*" or "patient-physician relation*" or "patient-provider relation*" or "patient-clinician relation*").ti,ab,kf.
7. ("provider-provider relation*" or "team relation*" or "inter?professional relation*" or "inter?clinician relation*" or "care team relation" or "interprofessional collaboration*").ti,ab,kf.
8. trust.ti,ab,kf.
9. 1 or 2 or 3 or 4 or 5 or 6 or 7 or 8
10. exp Educational Technology/
11. exp Telecommunications/
12. technology transfer/
13. Culturally Appropriate Technology/
14. technology/
15. Information Technology/
16. exp Medical Records/
17. exp Informatics/
18. exp Communications Media/
19. exp Management Information Systems/
20. ((communicat* or health* or informat* or comput* or medical or integrat*) adj3 (technol* or system* or applicat* or process*)).ti,ab,kf.
21. ((informat* or communicat*) adj3 (exchang* or tech*)).ti,ab,kf.
22. (electronic adj3 record*).ti,ab,kf.
23. (ehealth or e-health or electronic health or telehealth or tele-health or telemedicine or tele-medicine or tele-nursing or telenursing or telecommunicat* or tele-communicat* or digital health or videoconferenc* or video-conferenc* or virtual care or teleradio* or teleradio* or telemetry or mobile app* or smartphone* or informatics or mobile health or mhealth or m-health or software or EHR? or EMR?).ti,ab,kf.
24. ((virtual or remote* or distance or distant or online or mobile or video or asynchronous) adj3 (consult* or health or healthcare or medicine)).ti,ab,kf.
25. exp Telemedicine/
26. 10 or 11 or 12 or 13 or 14 or 15 or 16 or 17 or 18 or 19 or 20 or 21 or 22 or 23 or 24 or 25
27. exp Primary Health Care/
28. (clinic* or practi* or primary or physician* or refer* or visit* or outpatient* or consult* or family or communit* or ambulatory or centre? or center? or office).ti,ab.
29. 27 or 28
30. exp Meta-Analysis as Topic/
31. Review Literature as Topic/
32. Systematic Review/
33. (meta analy$ or metaanaly$ or systematic review$ or systematic overview$ or scoping review$ or umbrella review$).ti,ab,kf.
34. Systematic Reviews as Topic/
35. (cochrane or embase or psychlit or psyclit or psychinfo of psycinfo or cinahl or cinhal or science citation index or scopus or web of science or bids or cancerlit or ageline).ab.
36. (reference list$ or bibliograph$ or hand-search$ or relevant journal$ or manual search$).ab.
37. 30 or 31 or 32 or 33 or 34 or 35 or 36
38. 9 and 26 and 29
39. 37 and 38
40. limit 39 to english language
41. limit 40 to yr="2008 -Current"
