# Supplementary material for: The impact of eHealth on relationships and trust in primary care: a review of reviews
Source: BMC Prim Care. 2023 Nov 3;24:228. doi: 10.1186/s12875-023-02176-5 (PMC10623772; doi:10.1186/s12875-023-02176-5)
Supplement: Supplementary file 2 — Additional file 2. Impact of technology on patient-provider relationships. [file 12875_2023_2176_MOESM2_ESM.docx]

**Additional file 2: Impact of technology on patient-provider relationships**

| **Study name/authors** | **Patient-related factors** | **Provider-related factors** | **Technology-related factors** | **Other factors** | **Impact on relationships, trust, and/or related aspects** | **Associated impacts** |
| --- | --- | --- | --- | --- | --- | --- |
| ***Management systems*** | | | | | | |
| Shachak et al. (2009) | None reported | Provider communication skills and technology use style (screen gaze, ability to navigate the computer, baseline communication skills, spatial organization) | None reported | None reported | Mixed impact on rapport building and communication (mostly negative on rapport, related to provider screen gaze and computer-driven information gathering; sometimes positive influence on verbal and non-verbal communication)  Negative impact can be overcome by improvement in provider’s communication style and technology use | Patient-centredness, patient satisfaction with physician’s familiarity, communication about medical issues, and comprehensiveness of medical decisions |
| Irani et al. (2009) | Patient perception (of provider qualifications/level of experience) | Provider qualifications/level of experience | None reported | None reported | Mixed impact:  Negative impact on clinical interaction and relationship (patients seeing trainees/residents less likely to be satisfied with their relationship than patients seeing faculty)  No impact on time available for patient concerns, communication about psychosocial issues, attention given to the patient and family. | None reported |
| McGinn et al. (2011) | Patient concerns and perceptions (around hearing bad news electronically or providers using EHR to select patients) | Provider concerns and perceptions (around EHR reducing time spent with patients and interrupting their ability to provide direct care) | None reported | None reported | Mixed, but mostly negative impact on relationships (negative impact related to provider and patient concerns while some patients perceived no impact) | Reducing job performance |
| Bassi et al. (2012) | None reported | Provider perception (related to their use of technology) | None reported | None reported | Mixed impact on relationships and communication (generally positive views, with users having more positive views compared to nonusers) | None reported |
| Kazmi et al. (2013) | Patient perception (about role of technology) | Provider communication skills and technology use style (keyboarding, screen gaze) | None reported | None reported | Mixed impact on communication that is context-dependent (can amplify existing positive and negative communication, can positively impact information sharing for e.g., through patient perception that EHRs improve comprehensiveness of records, can negatively impact maintenance of a positive relationship, disrupt patient satisfaction, reduce trust e.g., through provider computer keyboarding and screen gaze, and limit patient engagement)  Negative effects can be mitigated through training, recommend considering patient experiences while implementing new technologies and be cognizant of potential adverse effects | None reported |
| Nguyen et al. (2014) | None reported | Provider technology use style (technology use within and outside the interaction, collaborating with patient to enter information into the EHR) | Technology design and features (being able to view imaging results with the provider) | None reported | Mixed impact on relationships (divert clinicians’ time from patient care to administrative tasks and attending to the computer screen, can create patient-focused interactions, improve collaborative access to information with provider) | None reported |
| Alkureishi et al. (2015) | None reported | Provider communication skills (e.g., eye contact, body postures, verbal and nonverbal cues of listening) and technology use style (e.g., keyboarding, computer use during in-person meetings) | None reported | None reported | Mixed impact on interaction and communication (provider behaviours can facilitate patient-centred interaction e.g., making computer use less obvious, cessation of computer use during sensitive discussions, inviting patients to look at the screen or negatively impact communication e.g., screen gaze, silence and close body posture) | None reported |
| Rathert et al. (2017) | None reported | Provider communication skills and technology use style (non-verbal behaviours like keyboarding, gazing, eye contact, facial orientation) | None reported | None reported | Mixed impact on communication and relationships (keyboarding, gazing, broken eye contact, indirect facial orientation can undermine relationship development and maintenance and interfere with provider’s information collection; patient portals and secure messaging can build trust, increase patient empowerment and engagement)  Recommend addressing concerns through provider training, improvement of processes and development of EHRs that capture useful data without interfering with communication | None reported |
| Mold et al. (2018) | None reported | None reported | None reported | None reported | Positive impact on relationships (improved patient self-care behaviours, shared disease management and decision-making, greater patient empowerment) and mixed impact on communication and interaction (patients valued “virtual presence” created through viewing results, asking for and sharing information, ability to communicate and engage with providers outside working hours; however, this also increased provider workload and time commitments for patient communication) | None reported |
| Diffin et al. (2019) | None reported | None reported | None reported | None reported | Positive impact on relationships (enhanced engagement, communication, access to information promoted “partnership in care”, made it easier to ask questions, increased confidence in initiating conversations, more comfortable to talk about sensitive issues) | Better multidisciplinary care and improved long-term outcomes |
| Lordon et al. (2020) | Patient expectations and perceptions (expectation to be involved with their data and for data to inform treatment plans, perception that use of PGHD would result in reduced face-to-face interaction) | Provider technology use style  Provider perceptions and concerns (perception that PGHD more useful for some patients and that patients preferred in-person interactions; concern that reviewing PGHD takes time away from clinical encounter, use of PGHD would exacerbate social isolation, would be challenging to diagnose without in-person cues) | Technology design and features (e.g., availability of trends and summary measures) | None reported | Mixed impact – positive impact on communication (related to providers’ use of PGHD as a discussion tool to provide emotional support and focus on issues pertinent to the patient and providers telling patients when to expect communication about their PGHD) and mixed impact on collaboration and relationships (supported when trends and summary measures were available and training was provided, and hindered because of insufficient data sharing capabilities, reduced time with patient and limited flexibility to meet patient-clinician needs; perceived negative impact also related to provider and patient perceptions and concerns about PGHD, provider perceptions of patient expectations)  Recommend setting clear expectations, improving visualization of data for clinicians through platforms or asking patients to summarize their data. | None reported |
| Benjamins et al. (2021) | None reported | None reported | None reported | None reported | Mixed impact – positive impact on communication (improved interaction, created level playing field, removed communication barriers, allowed caregivers to view notes from visits they couldn’t attend) and mixed impact on relationships (patients felt better about their doctor, appreciated their expertise, experienced equal relationship, valued transparency which contributed to trust, felt heard and cared for, but providers feared damage to relationship especially when notes revealed subjective impressions) | None reported |
| Tapuria et al. (2021) | None reported | None reported | None reported | None reported | Mixed but mostly positive impact on relationships and communication (better doctor-patient relationship, patient-provider communication, caregiver-provider communication, data becomes a “shared collaborative artifact” between patients and providers, some patient-reported concerns around potential for unauthorized access, prejudice, and misuse of data) | None reported |
| Wark et al. (2021) | None reported | None reported | Technology design and features (integration of social determinants of health into EHR) | None reported | Positive impact on relationships (by stimulating conversation, promoting shared decision-making, expanding social service referrals, helping identify unmet needs, and guiding the formation of community partnerships) | None reported |
| Zurynski et al. (2021) | None reported | None reported | Accessibility (of information to providers) | None reported | Positive impact on interaction (related to information accessibility) | None reported |
| Schwarz et al. (2022) | None reported | Provider technology use style (use of EHR during the appointment) | Accessibility (of provider notes to patients, referred to as “open notes”) | None reported | Mixed impact on relationships and trust (providers felt that open notes can positively impact collaboration, relationship, and communication, but may also create a disconnect and negatively impact facilitation of discussions and good relations during visits; service users felt that EHR use during appointments increased collaborative planning and documentation, but inaccurate documentation that did not match with their recollection of the visit negatively impacts transparency, respect and trust) | None reported |
| ***Communication systems*** | | | | | | |
| Verhoeven et al. (2010) | None reported | None reported | None reported | None reported | Mixed impact:  Positive impact on interactions but not significant compared to usual care (increased intensity of contact, providers perceived by patients as more supportive, improved data sharing).  Some studies reported negative impact on relationship and trust (ICT-based care thought to reduce the trusting and confidential relationship) | None reported |
| Brewster et al. (2013) | Patient perception (about technology) | Provider perception (about patient expectations and abilities) | Technical issues with equipment | None reported | Mixed impact on relationships – negative impact mostly perceived by nonuser providers (perceived shifting of focus away from patients with use of technology, equipment seen to inhibit conversation and relationship-building, believed that patients found equipment difficult to use, were too dependent on equipment, perceived transfer of responsibility to and limited trust in patients to self-monitor and make judgements about health-related matters) and positive impact perceived by user providers and patients (patients felt equipment reduced anxiety and improved self-management). | None reported |
| Simpson et al. (2014) | Patient diagnosis  Patient perception  Patient communication skills and participation (more active participation, initiative, spontaneity, trust, disinhibition) | Provider qualifications (therapist vs. physician)  Provider attitudes (towards videoconferencing)  Provider communication skills (adjusting during videoconferencing for e.g., being more deliberate and overt in nonverbal responses, asking more questions to clarify patient’s nonverbal behaviours, offering initial in-person sessions) | Care delivery modality (videoconferencing sessions compared to in-person therapy) | None reported | Mixed impact:  Mixed impact on therapeutic alliance (mostly rated in comparison to in-person therapy as equivalent, sometimes improved – potentially when therapists and patients adjust communication style and involvement during videoconferencing – and sometimes impaired – e.g., in epilepsy and post-traumatic stress disorder patients, by psychologists who are sceptical about building alliance via video)  Positive impact on bond (rated higher in-person, but also high in videoconferencing due to therapists’ ability to view themselves and progressed over time) including presence (clients and therapists forgot they were not in the same room, social presence rated stronger than physician presence) and empathy (clients felt less anxious and fearful about expressing emotions and more empowered to turn video off or leave) | None reported |
| Keijser et al. (2016) | Patient perception and expectations (perception that face-to-face meetings are fundamental to the relationship, expectations for timely responses from providers) | Provider perception of patient expectations (about adequacy of interpretation of shared information) | Task-technology fit | None reported | Mixed impact on relationships (information sharing could potentially improve shared decision-making but also lead to patient uncertainties due to inadequate interpretation, delays in email responses and scheduling mishaps can lead to patient frustration).  Recommend that physicians must judge appropriateness of modality according to the patient and situation and must be well-trained and knowledgeable about limitations and regulations related to technology | None reported |
| Petit et al. (2016) | None reported | None reported | None reported | None reported | Mixed impact on relationships (patients empowered with knowledge, become active partners and experts, but could result in knowledge competition between providers and patients) | None reported |
| Henry et al. (2017) | Patient sociodemographics (language barriers) | Provider’s cultural awareness and knowledge  Provider’s communication skills (e.g., use of nonverbal cues, questioning style)  Sociodemographics (language barriers) | Care delivery modality  Technical problems | None reported | Mixed impact:  Positive impact on making connections (increased cultural awareness and knowledge of resource availability facilitates making connections) and trust (increased feelings of safety through telehealth)  Mixed impact on communication (depending on provider’s ability to develop a “video presence” and “adjust to the medium” using nonverbal cues and appropriate communication style, but communication challenges exacerbated by technical problems and language barriers) | None reported |
| Richards et al. (2018) | None reported | None reported | None reported | None reported | Positive impact on communication and relationships (patients perceived increased access to specialists, amount of communication, and responsiveness of clinicians, reduced uncertainty and less “bothersome” to contact clinicians) | None reported |
| Watkins et al. (2018) | None reported | Provider communication skills and technology use style (direct communication through device and indirect communication via tailored text messages) | None reported | None reported | Positive impact (direct and indirect communication through device makes the patient feel cared for, makes them feel like they have empathetic support, prompts patient engagement with the health worker) | Increases motivation and leads to change in patient behaviour |
| Penny et al. (2018) | None reported | None reported | Care delivery modality (videoconferencing in comparison to phone and in-person) | None reported | Positive impact of videoconferencing compared to telephone consultations on communication and relationships (providers perceived increased closeness and understanding through sharing of non-verbal information, improved engagement, closeness, rapport, continuity) and negative impact compared to in-person consultations (reduced conversation flow, created challenges for providers to act “naturally” and discuss “sensitive” issues when caregivers present, prolonged relationship-building process) | None reported |
| Walker et al. (2019) | Patient perception | None reported | None reported | None reported | Mixed impact:  Negative impact on communication, interaction, and trust (related to patient perception that technology would replace personal care and human encounters and shift providers’ concern to data more than their symptoms and concerns)  Positive impact on roles within relationships (patients feel like an “equal partner” as they are able to discuss their monitoring data, supporting self-manxment and shared decision-making) | None reported |
| Qudah et al. (2019) | None reported | None reported | Technology design and features (personalized app design, realtime monitoring, two-way communication) | None reported | Positive impact on communication and relationships (improve information sharing and feedback, facilitate emotional presence by allowing therapists to demonstrate compassion and empathy, improve continuity of care, facilitate power and responsibility sharing, and increase patient trust in the provider) | None reported |
| Foong et al. (2020) | Patient perception | Provider perception  Provider technology use style | None reported | None reported | Mixed impact:  Positive impact on communication, shared decision making (use of digital technology to share physiological information, progress charts etc.), trust (based on patient perception of providers’ competence, knowledge and skills)  Negative impact on patient engagement (use of digital technology by care team to discuss care without the patient), communication (provider perception that digital tech reduces direct contact with patient) | None reported |
| Odendaal et al. (2020) | None reported | Provider perceptions (about the need for face-to-face contact, level of patient ownership, access, being contactable outside work hours, need for boundary setting) | None reported | None reported | Mixed impact on relationships, communication, and interaction (depending on provider perception for e.g., some perceived increased access to services through mHealth whereas others wanted in-person contact, had concerns with “impersonalisation” of interactions, and felt the need to set boundaries) and on trust (patient trust in device translated to trust in the service) | None reported |
| Vimalananda et al. (2020) | None reported | None reported | None reported | None reported | Negative impact on communication (lost opportunities for questions and conversations with specialists that might occur during in-person visits, but might be acceptable by patients in certain situations) | None reported |
| Wehmann et al. (2020) | None reported | None reported | Care delivery modality (technology-based vs. face-to-face interventions) | None reported | No impact on therapeutic alliance (ratings did not differ for technology-based and face-to-face interventions) | None reported |
| Wickramasekera et al. (2020) | None reported | None reported | None reported | None reported | Positive impact on communication (enhance information exchange, facilitate more time for communication and shared decision-making) and partnership and rapport building (patients more likely to express and clinicians more actively elicit cues and emotional concerns) | None reported |
| LeBlanc et al. (2020) | Patient diagnosis  Patient sociodemographics (age) | None reported | None reported | None reported | Mixed impact on relationships – positive (patients perceived increased rapport from regular contact, good connection with caregivers as they could be present, improved consistency and continuity, increased willingness and comfort with sharing information virtually particularly for mental health concerns and teenagers) and negative (providers perceived challenges with reading emotional cues while breaking bad news, need for human contact to facilitate recovery, particularly in mental health) | None reported |
| Irvine et al. (2020) | Normative understanding and expectations (of “typical” therapy session vs. phone session – unclear if this is driven by patient or provider) | | Care delivery modality (face-to-face vs. phone) | None reported | Mixed impact on alliance (alliance established over phone “different” from face-to-face, greater task/treatment focus of therapist can compensate for reduction in bond, makes it easier to stick to time boundaries visual anonymity over phone beneficial) | None reported |
| Thiyagarajan et al. (2020) | Patient perceptions and concerns (around privacy, sharing sensitive information and technological issues during videoconferencing) | Provider perceptions and concerns (around quality of physical exams, ability to choose appropriate investigations, assess mental health patients and access for disadvantaged and vulnerable groups with videoconferencing) | None reported | None reported | Mixed impact (patients felt videoconferencing is patient-centred and it is possible to build rapport, but both clinicians and patients prefer face-to-face because of their concerns around videoconferencing) | None reported |
| Gorrie et al. (2021) | Patient perception | None reported | None reported | None reported | Mixed impact on rapport – Negative impact perceived and anticipated by some patients (because of difficulty in responding to verbal cues and body language), but actual rapport found to be satisfactory after the virtual appointment by most patients. | Limitations of telegenetics result in patients feeling like they receive less emotional support and not having their psychosocial needs met |
| Siegel et al. (2021) | Patient diagnosis (presence of cognitive or behavioural problems)  Patient sociodemographics (socioeconomic status) | None reported | Care delivery modality (phone vs. videoconferencing) | None reported | Negative impact on communication (providers find that telephone use is preferred by patients of lower socioeconomic status, but feels less personal, challenging to collect information and maintain therapeutic alliance in the absence of facial and body cues; remote delivery creates challenges in focusing in the presence of interruptions and distractions especially for patients with cognitive behavioural challenges; videoconferencing can lead to loss of boundaries when patients unintentionally access personal information about providers’ homes, and patient and provider burnout from overuse)  Suggest providing training for providers on using telehealth and modifications for telephone delivery | None reported |
| Dalley et al. (2021) | None reported | Provider communication skills (interprofessional talk and clarification) | None reported | None reported | Negative impact on patient role within the relationship (prolonged periods of interprofessional talk and clarification can limit patient participation in team-based care) | None reported |
| Keenan et al. (2021) | None reported | None reported | None reported | None reported | Mixed but mostly negative impact on relationships and trust (telehealth can create a lack of human touch and distance, crucial role of face-to-face methods in healing, undermining of trust and respect through telehealth especially if patient and provider have never met before, need for additional actions to win trust, privacy and confidentiality concerns, but can reduce loneliness and provide more protected setting where patients feel likely to be listened to)  Recommended that context and patient preferences be considered when designing services | None reported |
| Drovandi et al. (2021) | Patient sociodemographics (age) | None reported | None reported | None reported | Positive impact on communication (clinicians found telehealth to be effective for communicating with patients; patients, particularly older patients, felt that telehealth facilitated discussions with providers and supplemented standard visits) | Holistic and individualized care |
| Ferguson et al. (2021) | Familiarity and consistency within relationship | | None reported | None reported | Mixed impact on communication and relationships (patients and providers felt that telemonitoring facilitated and enhanced communication and reliable interaction, more regular and effective communication was noted when the provider remained the same, some patients and providers preferred face-to-face contact for developing a therapeutic relationship) | None reported |
| Howard et al. (2021) | None reported | None reported | Care delivery modality (face-to-face vs. videoconferencing) | None reported | No impact (no difference in therapeutic alliance between care delivered face-to-face and via videoconferencing, suggesting that remote delivery methods do not hinder alliance) | None reported |
| Kinley et al. (2021) | Patient sociodemographics (age)  Presence of pre-existing relationship | Presence of pre-existing relationship | Care delivery modality (remote vs. in-person)  Technology design and features (screen sharing, editing documents) | None reported | Positive impact on information and support provision and shared decision-making (providers can deliver individualized information and advice and provide timely support and advice via remote consultations, patients can understand and engage in collaborative discussions)  Positive impact on relationships and communication (presence of pre-existing relationship enables providers to engage patients in shared decision-making and self-management via remote consultation; remote consultations facilitate consistent access to same provider and lead to creation and sustenance of positive working relationships, particularly in younger patients; specific technology features promote collaborative discussions and effective communication via videoconsultation) | Positive patient outcomes including increased patient understanding, improved control over their condition, increased quality of life, greater self-efficacy, regular review for patients who may not attend face-to-face sessions, more shared decision-making, more discussion of personal preferences, increased attendance at reviews, more engagement and increased confidence |
| Sharma et al. (2021) | None reported | Provider perception and experience of using technology | None reported | None reported | Mixed impact (providers concerned about potential negative impact of telepsychiatry on rapport, but perceived positive impact after use; patients consistently reported satisfaction whereas providers reported concerns about “translation of doctor-patient relationship to the screen”) | None reported |
| Spelten et al. (2021) | Patient sociodemographics (age, computer literacy, minority status)  Presence of pre-existing relationship | Presence of pre-existing relationship | Care delivery modality (telephone vs. videoconferencing) | None reported | Mixed impact on relationships, trust and communication (young computer literate people reported being able to develop a trusting relationship with their provider via telehealth compared to older people; patients and providers perceived limited access to non-verbal cues and capacity for relationship-building via phone, particularly for minority participants, whereas videoconferencing was perceived to facilitate non-verbal communication and provide reassurance; positive experiences reported more often when phone and video consultations occurred concurrently; patients mostly reported positive experiences with telehealth when it facilitated maintenance of pre-existing relationship) | None reported |
| Verma et al. (2021) | Patient sociodemographics (language barriers, more commonly noted with patients in high social vulnerability index areas)  Patient diagnosis (hearing impairments)  Presence of pre-existing relationship | Presence of pre-existing relationship | None reported | None reported | Negative impact on communication and rapport (providers reported challenges for patients in expressing themselves and communicating, particularly when there were language barriers or hearing impairments; providers and patients felt that a lack of physical touch made it difficult to build bonds with new patients; patients found telemedicine impersonal even when they knew their provider) | None reported |
| Wallace et al. (2021) | Patient perception and expectations (regarding therapeutic touch) | None reported | Care delivery modality (face-to-face vs. telehealth) | None reported | Mixed impact, but mostly positive on therapeutic alliance (some patients found telehealth impersonal while others began to find it more personal after overcoming initial scepticism, perceived more individual attention and focus compared to face-to-face consultations; providers felt it was possible to foster rapport and trust via remote consultations and found that it provided more time for listening and refining verbal cues and language to personalize the experience and develop a better relationship) | None reported |
| Wu et al. (2021) | None reported | None reported | Care delivery modality (audio-only vs. video vs. face-to-face visits) | None reported | Mixed impact on relationships with overall positive impact as perceived by patients (some providers and patients reported high connectedness, found virtual visits similar to face-to-face and perceived greater family inclusion and support during virtual visits; others wanted more opportunities to connect, perceived less compassion and empathy, were uncomfortable about potential for multiple people to watch during virtual visits, wished to see provider’s reaction and perceived inadequate time for questions during audio-only visits)  The authors note that none of the included studies explicitly explored how telemedicine impacts patient-provider relationships and suggest the need for more studies investigating “how virtual visits could augment patient-provider relationships” | None reported |
| de Albornoz et al. (2022) | Presence of pre-existing relationship | | Care delivery modality (face-to-face vs. telephone vs. video consultations) | None reported | Mixed impact:  Positive impact on therapeutic alliance (high therapeutic alliance over teleconsultation)  Mixed impact on communication and relationships (positive impact when there is a pre-established relationship, richer information provision and advice in face-to-face visits compared to teleconsultations, comparable interaction in terms of content and quality between telephone and video consultations, better rapport building in video consultations, less effective communication in phone consultations due to limited access to non-verbal cues) | Better treatment continuity and clinical outcomes (when there is an established relationship between patient and provider) |
| Walthall et al. (2022) | Patient attitude  Patient diagnosis (stage of illness) | Presence of pre-existing relationship  Provider communication style (undivided attention, supportive and friendly environment, feeling less rushed)  Provider perception and experience of using technology | None reported | None reported | Positive impact on relationship and rapport building (rapport building facilitated by presence of pre-existing relationship but not necessarily negatively impacted by its absence; patients found that provider communication style facilitated relationship building, providers found that patient receptiveness facilitated positive relationships, provider perceptions of impact of remote consultations on relationship and rapport building changed from positive to negative post-implementation)  Positive impact on roles within relationship (patients and providers felt that remote consultations facilitated and empowered patient self-management and increased active patient participation but noted that this might change as their illness progresses)  The authors recommend communication skills training for clinicians to conduct remote consultations. | None reported |
| Diaz et al. (2022) | None reported | None reported | None reported | None reported | Negative impact on rapport, communication, and relationships (lack of comfort and rapport with providers in a virtual setting, virtually mediated communication considered  “impersonal”, harder to build a trusting relationship with provider | None reported |
| Lampickiene et al. (2022) | None reported | None reported | None reported | None reported | Mixed impact on interaction, relationship and rapport (digital visits sometimes seen as more personal and intimate and sometimes less due to lack of physical presence and difficulty maintaining patient’s attention/engagement; challenging to connect and manage emotional situations due to limited non-verbal information and inability to use conflict management techniques remotely; could form strong alliances or cooperation with therapists, possible to build rapport and have an authentic relationship with patients)  The authors note that virtual visits can complement rather than replace in-person visits where “social interaction and physical presence facilitate better conditions for showing empathy and simply being there for the patient”. | None reported |
| Lindenfeld et al. (2022) | Patient sociodemographics (language)  Patient diagnosis (visual or auditive impairments) | None reported | None reported | None reported | Mixed impact on relationships and communication (overall positive impact with telemedicine enabling access to home-bound patients and involving family members; some potential for telemedicine to decrease “human connection” as it makes it less feasible to convey empathy and can create communication barriers with patients speaking non-native languages or with visual or auditive impairments)  The authors recommend “interpersonal training specific to telemedicine” for providers to “preserve the relational aspect of primary care”. | None reported |
| ***Computerized Decision Support systems*** | | | | | | |
| Scalia et al. (2019) | None reported | None reported | None reported | None reported | Positive impact on communication (both patients and clinicians perceived improved information exchange and shared decision-making, observational data also indicate change in communication patterns and increase in shared decision-making) and no impact on trust | None reported |
| Yen et al. (2021) | Patient sociodemographics (socially disadvantaged populations) | None reported | None reported | None reported | Positive impact on communication (for patient decision aids tested among socially disadvantaged populations) | None reported |
| Čartolovni et al. (2022) | None reported | Provider communication skills (transparency and clear communication with patients about limitations and performance gaps) | Technology features (safety and reliability of tools) | None reported | Mixed impact on relationship and trust (not disclosing the use of an AI-based decision support tool can negatively impact relationship, patient autonomy and trust; may transform bilateral relationship into a trilateral one by creating “computernalism” and reducing physician involvement and personal interaction during decision-making, but can also provide more time for providers to engage in empathetic, compassionate, and trusting relationships by improving diagnostic and prognostic efficiency) | None reported |
| ***Information systems*** | | | | | | |
| Farnood et al. (2015) | Patient motives and perceptions | Provider perception and reaction or response to information | None reported | None reported | Mixed impact:  Positive impact on relationships (related to patients’ motives to support rather than challenge therapeutic relationship, providers able to create an open environment that encourages patients to share information, provider honesty about their knowledge, provider perception that patients have right to be informed), roles within relationship (related to patient and provider perception that patient is more involved and provider acts as partner rather than authority), communication and interaction (patients feel informed and prepared for appointments, making interactions more respectful, interactive, efficient and effective)  Negative impact on relationships and trust (related to providers perceiving internet health information as damaging to the relationship, providers feeling the need to address often misleading or inaccurate information, providers’ negative reaction to information, provider perception that patients don’t trust them, provider anxiety when information is outside their area of expertise) | None reported |
| Luo et al. (2022) | Patient attitudes (towards online health information, willingness to discuss information with provider) | Provider attitudes (towards patients sharing online health information with them) | Quality of online health information | None reported | Mixed impact on relationships (greater perceived positive impact on communication and decision-making in patients who value online health information and consider it helpful for decision-making; good quality information and patient willingness to discuss information with provider positively impacts relationship while “uneven quality” information, patient fear of challenging provider authority and provider trying to maintain their authority when patients share information can negatively impact relationship) | None reported |
| ***Multiple technologies*** | | | | | | |
| Crooks et al. (2008) | None reported | None reported | None reported | None reported | Positive impact on relationships suggested when providers communicate with patients to clarify information needed for effective care, as this can assist with creating a “relationship of information-sharing” | None reported |
| Ludwick et al., (2009) | None reported | Provider communication skills and technology use style | Spatial organization of technology (computer monitor placement, physical space available for systems) | Organizational factors (implementer concerns about impact of implementing health information systems on patient-provider relations) | Mixed impact (implementer concerns about potential negative impact which can be managed through sound project management, strong leadership, implementation of standardized terminologies and staff training; positive impact related to providers turning away to enter data creating time for patient reflection and enhancing information transfer, showing patients their record) | None reported |
| Kruse et al. (2015) | None reported | None reported | None reported | None reported | Mixed impact on communication | None reported |
| Barbosa et al. (2016) | None reported | Provider communication skills (absence of visual information, needing to rely upon descriptions of patients) | None reported | None reported | Negative impact on relationships and trust (absence of visual information can impair development of relationships of trust) | None reported |
| Crampton et al. (2016) | None reported | Provider technology use style | None reported | None reported | Mixed impact on interaction (provider computer use moves the interaction from a narrative to a checklist-oriented conversation, but clinicians employ strategies to maintain patient-centredness in computerized settings) | None reported |
| Clarke et al. (2016) | None reported | None reported | None reported | None reported | Positive impact on communication reported in one included study. | None reported |
| Patel et al. (2016) | None reported | Provider communication skills and technology use style (including computer use, conversational style, room design, non-verbal skills and posture) | None reported | None reported | Mixed impact on interpersonal interactions and shared decision-making (depending on provider use for e.g., using the computer to facilitate conversation, maintaining eye contact, maintain conversation or paraverbal expressions, facing patients are facilitators while “going against these guidelines” can be a barrier) | Cognitive-affective outcomes |
| Rouleau et al. (2017) | None reported | None reported | None reported | None reported | Positive impact on relationships (create pathways for communications, new types of bonds with patients, establish trust through videoconferencing and sense of connection from patient perspective) | None reported |
| Adjekum et al. (2018) | Stakeholder motives (altruism)  Stakeholder perceptions or experiences (ease of use, self-efficacy, usefulness, excessive costs, fear of data exploitation)  Sociodemographic factors  Fair data access  Recommendation from family members, acquaintances and colleagues  Limited accessibility | | Technology design and features (customizable design, interoperability, privacy)  Technical problems (defective technology) | Organizational factors (labelled as institutional elements by authors – e.g., guidelines for standardized use, stakeholder engagement, improved communication, decreased workloads, insufficient training, service provider reputation, inadequate publicity) | Mixed impact on patient trust in technology depending on whether factor is an enabler, impediment or both (for e.g., ease of use influences trust in digital health systems positively, fear of data exploitation influences trust negatively, and sociodemographic factors can influence trust either positively or negatively) | None reported |
| Palacholla et al. (2019) | None reported | None reported | None reported | None reported | Mixed impact on communication and relationships (with some patients perceiving improved direct communication with provider, feedback and participation in shared decision-making and others concerned with impact of reduced in-person contact). | None reported |
| Davies et al. (2020) | None reported | Provider concerns and perceptions (about establishing and maintaining relationships, about modality) | Care delivery modality | None reported | Mixed impact on therapeutic relationships (some concerns about negative influence on rapport but mostly perceived as “different” but “not necessarily worse” and some surprised by their ability to develop relationships online, blended treatments provide more opportunities for rapport, support, monitoring) | None reported |
| Sunjaya et al. (2020) | None reported | None reported | None reported | None reported | No impact on relationship (quality of relationship remains similar during telepsychiatry and face-to-face therapy) | None reported |
| Hilty et al. (2021) | None reported | None reported | Care delivery modality (asynchronous compared to synchronous technology and in-person interaction) | None reported | Mixed impact on therapeutic engagement and presence (synchronous is functional for a quick engaging discussion but not exactly like being in-person; asynchronous can facilitate strong alliance with quick exchanges over time, expand communication, continuity and connection, despite the absence of verbal and non-verbal cues and limited social connectedness) and trust (immediacy and interaction builds trust, but can lead to users assuming others’ intentions and inadequate investigation of potential negative consequences)  Suggest strategies for care delivery according to modality (e.g., use words for a patient in tears instead of offering a tissue) | None reported |
| Noblin et al. (2021) | User concerns (related to data confidentiality and security, parents and children accessing each other’s information, uncertainty about and distrust in functionality) | None reported | None reported | None reported | Potential negative impact on informal caregiver trust in technology (related to user concerns) | None reported |
| Al-Naher et al. (2022) | None reported | None reported | Technology features (opportunities for communication using the technology) | None reported | Mixed impact on communication (interventions that facilitated human contact and provided communication opportunities perceived to have a positive impact by patients and providers, such as reducing patients’ feelings of isolation, increasing trust between providers and patients, and providers perceiving patients to be more open; with interventions that led to reduced contact and communication, patients missed human contact and patients and providers perceived a “distance”) | None reported |
| Giordan et al. (2022) | None reported | None reported | Technology design and features (mobile apps allowing for data sharing and integration with EMR) | None reported | Positive impact on communication (mobile tools providing data sharing and EMR integration features considered “an excellent tool for communicating with clinicians”) | None reported |
| Hartasanchez et al. (2022) | None reported | None reported | Technology design and features (ease of use) | Training in technology use  Broadband access | Positive impact (can facilitate “partnership” and “complex interactions” necessary for remote decision-making and synchronous digitally mediated visits if factors like access to broadband, training in and ease of use of technology are addressed) | None reported |
| Shah et al. (2022) | None reported | None reported | Care delivery modality (phone vs. videoconferencing vs. face-to-face)  Quality of online health information | None reported | Mixed impact on relationships and trust (patients and providers experienced enhanced relationships, especially with telerehabilitation and real-time videoconferencing; patients valued “undivided communication” via phone-based interventions, but were concerned about the lack of in-person and visual contact; incomplete or misleading information on social media could potentially have a negative impact on patient trust) | None reported |
