# Supplementary material for: The impact of eHealth on relationships and trust in primary care: a review of reviews
Source: BMC Prim Care. 2023 Nov 3;24:228. doi: 10.1186/s12875-023-02176-5 (PMC10623772; doi:10.1186/s12875-023-02176-5)
Supplement: Supplementary file 3 — Additional file 3. Impact of technology on provider-provider relationships. [file 12875_2023_2176_MOESM3_ESM.docx]

**Additional file 3: Impact of technology on provider-provider relationships**

| **Study name/authors** | **Provider-related factors** | **Technology-related factors** | **Other factors** | **Impact on relationships and/or related aspects** | **Associated impacts** |
| --- | --- | --- | --- | --- | --- |
| ***Management systems*** | | | | | |
| McGinn et al. (2011) | Provider perception of technology | None reported | None reported | Negative provider perception of EHR systems as “management control mechanisms” could infringe on privacy and autonomy | None reported |
| Bassi et al. (2012) | Provider technology use skill (those with higher skill level of use of medical packages saw greater benefit from communication with other medical organizations) | None reported | Size of practice  (larger practices saw greater benefit for communication with other medical organizations and with fellow general practitioners) | Positive impact on communication (perceived positive effect of EMR on communication with other providers and organizations by both users and non-users; extent of benefit influenced by skill level of provider and size of practice) | None reported |
| Nguyen et al. (2014) | None reported | None reported | None reported | Mixed impact on communication (improved provider-provider communication within care team but negatively impacted between clinical areas and outside the system; adoption of EHR can lead to increased need for interdisciplinary communication) | None reported |
| Diffin et al. (2019) | None reported | None reported | None reported | Positive impact on information sharing (increased sharing of medical information across providers in the hospital system) | Improved clinical awareness of patients’ complexity and treatments |
| Wisner et al. (2019) | Technology use style (use of copy-paste function in EHR) | Technology design and features (structure, organization, volume of information, ease of information retrieval) | None reported | Negative impact on communication (templated notes, lack of quality and quantity of narrative notes, lack of ease in information retrieval, and frequent use of copy-paste function created challenges in communicating, learning about others’ thought processes, and developing “shared understandings”; lack of appropriate representational structures within EHR for nurse, patient and psychosocial perspectives of care led to fewer opportunities to share this information within the team or to team members not reading or “skimming” over this information) | None reported |
| ***Communication systems*** |  |  |  |  |  |
| Nguyen et al. (2015) | None reported | Technology features (e.g., unidirectional paging systems and systems that send “simultaneous notification across the medical hierarchy” can impair communication) | None reported | Mixed impact (increased efficiency and accuracy of information exchange and improved collaboration between providers but can also lead to more frequent interruptions and disruptions, reduce frequency and limit time for face-to-face interactions, and impair communication e.g., unidirectional systems that don’t allow for response) | Provider frustration |
| Keijser et al. (2016) | Provider communication skills (technical communication skills like timely response to electronic inquiries, virtual meeting skills, adherence to norms for virtual meetings; socioemotional processes (e.g., non-verbal cues, informal contact frequencies) | Fit between task and technology (e.g., selecting technology for communication that fits task) | Organizational factors (resource availability including standards and guidelines, training, strategic and creative adaptations) | Mixed impact on team relationships and communication (resource availability is vital for facilitating virtual team operations, team dynamics, task delegation; delayed email responses can generate friction, working relationships in virtual teams are weaker because of the lack of non-verbal cues and informal contact frequencies that foster relationship building and misunderstandings in communication can occur due to lack of close interpersonal contact) | None reported |
| Petit et al. (2016) | None reported | None reported | None reported | Mixed impact (creates “new alliances and conflicts” between providers, providers distribute patient care tasks “leading to more mutualization and less autonomy of professionals”) | None reported |
| Penny et al. (2018) | None reported | None reported | None reported | Positive impact on connection, information sharing and support (improved connection with colleagues, reduced isolation, provided opportunities for advice, information, and support) | None reported |
| Foong et al. (2020) | None reported | None reported | None reported | Positive impact on communication and collaboration (improved communication and collaboration among colleagues, can facilitate dialogue and reflection between specialist and non-specialist providers) and mixed impact on roles (can expand or diminish HCP roles within care team e.g., telemedicine nurses took on new roles like coordination, advocacy but only physicians were involved as the sole decision maker) | None reported |
| Odendaal et al. (2020) | Provider communication i.e., nature of interaction (i.e., type of response from supervisor) | None reported | None reported | Mixed impact on communication and interaction between lower and higher-level colleagues (improved connection, reporting, identifying and receiving support, but negative response from supervisor led to negative impressions about technology and reluctance to contact) | None reported |
| LeBlanc et al. (2020) | None reported | None reported | None reported | Mixed impact on relationships (decreased isolation, increased professional support from specialists, created network of providers for support and guidance, increased team meetings, strengthened relationships and communication between agencies and sectors e.g., local and urban hospitals, but in some instances led to disagreements between providers at different sites e.g., disagreement between local providers and specialists/urban doctor on recommendations) | Improved best practice and patient care  Patient distrust in providers at both sites |
| Vimalananda et al. (2020) | None reported | None reported | None reported | Positive impact on communication (can enhance primary care provider-specialist communication) | None reported |
| Dalley et al. (2021) | Provider communication skills (clarification actions and directional feedback i.e., allowing professionals to exchange relevant information regarding focus of the medical tasks) | None reported | None reported | Mixed impact on collaboration and communication (clarification actions can enhance collaborative working among health care professionals, directional feedback supports interprofessional communication) | Clarification actions can also limit patient participation |
| Siegel et al. (2021) | None reported | None reported | None reported | Negative impact on communication (insufficient communication due to loss of in-person interactions) | None reported |
| Drovandi et al. (2021) | None reported | None reported | None reported | Positive impact on communication (providers perceived more efficient communication through electronic documentation and interactive web-based platforms, reducing the need for unnecessary phone calls) | Smoother workflow |
| Lampickiene et al. (2022) | None reported | None reported | None reported | Negative impact (remote consultations and remote work can lead to feelings of isolation and loneliness from not being able to meet peers)  Recommend using online social activities like virtual social groups, peer support and games to create informal communication opportunities to replace in-person interactions | None reported |
| Lindenfeld et al. (2022) | None reported | None reported | None reported | Negative impact (synchronous telemedicine led to feelings of isolation in the absence of in-person interactions with colleagues) | None reported |
| Walthall et al. (2022) | None reported | None reported | None reported | Mixed impact on communication (some studies noted challenges to communicating between staff when using remote consultations, while others reported increased trust and improved team communication) | None reported |
| ***Multiple technologies*** |  |  |  |  |  |
| Barr et al. (2017) | Providers’ time  Provider attitudes and concerns (willingness to learn, concerns about confidentiality and privacy concerns) | None reported | None reported | Negative impact on professional roles and responsibilities and professional culture (conflict about roles and responsibilities, differing perceptions and experiences of power between different professional groups like telehealth nurses and in-person nurses/physicians) and positive impact interprofessional collaboration (online communities/learning resource to facilitate collaborative practice among providers can enhance information exchange and communication among professionals by bridging geographical distances, enabling interactions across institutions and professions, and providing motivation; however providers’ time, willingness to learn and confidentiality and privacy concerns can be barriers to this process) | None reported |
| Rouleau et al. (2017) | None reported | None reported | None reported | Positive effects on intra and interprofessional collaboration (improved communication between members of interdisciplinary team), relationships (better and more trustworthy relationships between nurses and doctors) and frequency of collaboration (more frequent collaboration between members of healthcare team). | None reported |
| Al-Naher et al. (2022) | None reported | Technology features (e.g., availability of features that allowed physicians to connect with specialist consultants) | None reported | Mixed impact on communication (providers felt that interventions that “seamlessly connect multidisciplinary teams” leads to positive team communication and encourages teamwork, whereas systems that did not connect with other team members negatively impacted teamwork) | None reported |
